# Effectiveness of outpatient and community treatments for people with a diagnosis of ‘personality disorder’: systematic review and meta-analysis

**DOI:** 10.1186/s12888-022-04483-0

**Published:** 2023-01-21

**Authors:** Panos Katakis, Merle Schlief, Phoebe Barnett, Luke Sheridan Rains, Sarah Rowe, Steve Pilling, Sonia Johnson

**Affiliations:** 1 Division of Psychiatry, University College London, London, UK; 2 NIHR Mental Health Policy Research Unit, Division of Psychiatry, University College London, London, UK; 3 Centre for Outcomes Research and Effectiveness, Research Department of Clinical, Educational and Health Psychology, University College London, London, UK; 4 National Collaborating Centre for Mental Health, Royal College of Psychiatrists, London, UK; 5 Camden and Islington NHS Foundation Trust, London, UK

**Keywords:** Personality disorder, Psychotherapy, Psychological interventions, Community treatments, Systematic review, meta-analysis

## Abstract

**Background:**

Quality of care and access to effective interventions have been widely criticised as limited for people diagnosed with ‘personality disorder’ or who have comparable needs (described in some recent papers as “Complex Emotional Needs” (CEN). It is important to identify effective interventions and the optimal context and mode of delivery for people with CEN. We aimed to investigate the effectiveness of psychosocial interventions delivered in community and outpatient settings in treating symptoms associated with ‘personality disorder’, and the moderating effects of treatment-related variables.

**Methods:**

We systematically searched MEDLINE, EMBASE, PsycINFO, CINAHL, HMIC, ASSIA for articles published in English, from inception to November 23, 2020. We included randomized controlled trials examining interventions provided in community or outpatient settings for CEN. The primary outcome was ‘personality disorder’ symptoms, while secondary outcomes included anxiety symptoms, depressive symptoms, and global psychiatric symptoms. Random-effects meta-analysis was conducted for each outcome, and meta-regression analysis was performed to assess the moderating effects of treatment characteristics. The quality of the studies and the degree of publication bias was assessed.

**Results:**

We included 54 trials (*n* = 3716 participants) in the meta-analysis. We found a large effect size (*g* = 0.78, 95% CI: 0.56 to 1.01, *p* < 0.0001) favoring interventions for ‘borderline personality disorder’ (BPD) symptoms over Treatment as Usual or Waitlist (TAU/WL), and the efficacy was maintained at follow-up (*g* = 1.01, 95% CI: 0.37 to 1.65, *p* = 0.002). Interventions effectively reduced anxiety symptoms (*g* = 0.58, 95% CI: 0.21 to 0.95, *p* = 0.002), depressive symptoms (*g* = 0.57, 95% CI: 0.32 to 0.83, *p* < 0.0001), and global psychiatric symptoms (*g* = 0.50, 95% CI: 0.35 to 0.66, *p* < 0.0001) compared to TAU/WL. The intervention types were equally effective in treating all symptom categories assessed. Treatment duration and treatment intensity did not moderate the effectiveness of the interventions for any outcome.

**Conclusions:**

People with a ‘personality disorder’ diagnosis benefited from psychological and psychosocial interventions delivered in community or outpatient settings, with all therapeutic approaches showing similar effectiveness. Mental health services should provide people with CEN with specialised treatments in accordance with the availability and the patients’ preferences.

**Supplementary Information:**

The online version contains supplementary material available at 10.1186/s12888-022-04483-0.

## Introduction

Difficulties associated with a ‘personality disorder’ diagnosis are common and debilitating, with the prevalence estimated to be 7.8% worldwide [1]. Reflecting the concerns around the diagnostic validity of the diagnostic term ‘personality disorder’ and the potentially stigmatising effect for those receiving this diagnosis [2–4], alternative terms, such as Complex Emotional Needs (CEN), have been developed to describe this condition [5]. In this review, the terms CEN and ‘personality disorder’ will be used interchangeably.

People diagnosed with ‘personality disorder’ experience severe impairment and distress from adolescence or early adulthood onwards, affecting various aspects of their life, including social and vocational functioning as well as mental and physical health [6–11]. Additionally, people with CEN experience poor well-being [12], lower quality of life [13], and reduced life expectancy [14], including an increased risk of dying from homicide, suicide, or accident compared to the general population [15]. Lastly, a diagnosis of ‘personality disorder’ is associated with high costs through high utilisation of healthcare systems, leading to increased economic and societal burden [16, 17].

Recent evidence suggests that the difficulties associated with a ‘personality disorder’ diagnosis change substantially over time, especially following psychological or psychosocial treatment targeting the core symptoms [18]. There is a consensus that psychological interventions are the first-line treatment for people diagnosed with ‘personality disorder’ [19, 20]. Α number of reviews and meta-analyses have found evidence that some types of community interventions, especially psychodynamically informed treatments and cognitive and behavioral therapy (CBT), are effective in treating ‘personality disorder’ symptoms [21–26].

The majority of studies have focused on ‘BPD’ and have shown that specialised psychotherapeutic interventions, such as psychodynamic therapy, dialectical behaviour therapy (DBT), mentalization-based therapy (MBT) and transference-focused therapy (TFT), are efficacious for this condition [27–35]. A recent Cochrane review found that a wide range of psychological interventions could effectively reduce ‘BPD’ symptoms, alongside other outcomes [32]. However, previous reviews have been limited in their inclusion criteria, focusing on samples with ‘BPD’ diagnosis, without examining people diagnosed with other ‘personality disorders’, such as ‘antisocial personality disorder’, ‘narcissistic personality disorder’ or cluster A and C ‘personality disorders’ or those reporting symptoms associated with CEN.

While a number of people experiencing CEN receive treatment in outpatient settings, including day hospitals, a substantial proportion of patients, predominantly those with a ‘BPD’ diagnosis, are admitted to inpatient hospitals where they receive short-term crisis interventions [36, 37]. Although inpatient treatment may sometimes be necessary and effective in treating acute symptoms associated with CEN [36, 38], the current focus is on the provision of interventions in community mental health care settings, including outpatient facilities, such as day hospitals, as the latter are beneficial for the establishment of long-term therapeutic outcomes [39], and cost effective [40].

The lack of definitive evidence regarding the optimal treatments for people with CEN has led to large heterogeneity in the guidelines across different countries [41], and the services provided for this population [42]. A further impediment lies in the focus of the guidelines on ‘BPD’ diagnosis, with only a few treatment recommendations for people diagnosed with other ‘personality disorders’ or experiencing associated experiences (e.g., repeated self-harm or suicide attempts, complex trauma or complex post-traumatic stress disorder (c-PTSD) [41]. Lastly, only limited evidence exists on the impact of the treatments’ qualities and the context that those are being provided (e.g., day hospital, generic mental health services), in the effectiveness of the Interventions.

In light of the above limitations, the aim of this systematic review and meta-analysis is to: a) examine the effectiveness of psychological and psychosocial interventions delivered in community settings in treating ‘personality disorder’ symptoms, as well as depressive, anxiety, and global psychiatric symptoms, b) compare the effectiveness of different intervention types for the outcomes above, and finally c) investigate the moderating effects of treatment characteristics in the effectiveness of the interventions, and the optimal context of delivery.

## Methods

This study was developed following PRISMA guidelines [43], and a protocol was registered on PROSPERO (reference: CRD42019143165). The current review was part of a programme of work that included four individual systematic reviews. A single search strategy was used for the whole programme. The protocol for the wider programme of work was also registered on PROSPERO (CRD42019131834). The current meta-analysis has its basis on a larger scoping review [44]. The extent of heterogeneity in the literature (e.g., samples included, outcomes assessed) led to the decision to conduct meta-analyses on a more limited subset of the data, including higher quality data from randomized control trials (RCTs) only.

### Search strategy and selection criteria

To identify eligible articles for this systematic review and meta-analysis, a search strategy was developed for the following bibliographic databases: MEDLINE, EMBASE, PsycINFO, Cumulative Index to Nursing and Allied Health Literature (CINAHL), Social Policy and Practice, Health Management Information Consortium (HMIC), and Applied Social Sciences Index and Abstracts (ASSIA). The original search, developed in collaboration with a project-specific working group that included people with lived experience of CEN, was conducted in December 2019. An update search was carried out in November 2020. The search encompassed terms relating to CEN, community or outpatient settings, and psychological or psychosocial treatments. Websites of known relevant organisations were also searched. Lastly, reference lists of all included studies were hand-searched, and reference search of relevant systematic reviews was conducted to identify further studies. Full details of the search strategy are shown in the Additional file 1 (Tables S1-S6).

Studies meeting the following criteria were included in this review:

**Population**: Adults (90% of the sample over 16 years old or a mean sample age of 18 or over) in which a majority (> 50%) was diagnosed with ‘personality disorder’ or participants identified as experiencing symptoms or difficulties related to a diagnosis of ‘personality disorder’ (i.e., repeated self-harm, emotion dysregulation or instability).

**Intervention**: Psychological or psychosocial interventions conducted in a community mental health care setting, including a wide range of outpatient facilities, such as day hospitals. Studies were included if participants were provided with any kind of group or individual psychological or psychosocial intervention, such as DBT, psychodynamic treatment, MBT or emotional regulation programmes. Interventions should primarily target CEN and follow a protocol developed for this population. We excluded interventions carried out in forensic, crisis care or inpatient settings.

**Comparator**: Eligible controls included TAU, standard care, WL or no intervention, or alternative types of active treatment(s).

**Outcomes**: Eligible outcomes included ‘personality disorder’ symptom severity or ‘BPD’ symptom severity, anxiety symptoms, depressive symptoms or psychiatric symptoms measured on a validated scale. In the instances that more than one rating scale was used to examine a specific outcome, one was chosen, based on the psychometric properties and the frequency of use in other included studies. Outcomes measured by subscales were excluded from the analysis, leaving only full scales developed for the specific outcomes.

**Study design**: Only RCTs were included in this review. Studies with alternative designs which met other criteria were included in our scoping review, which did not include a meta-analysis [44].

**Fig. 1:**
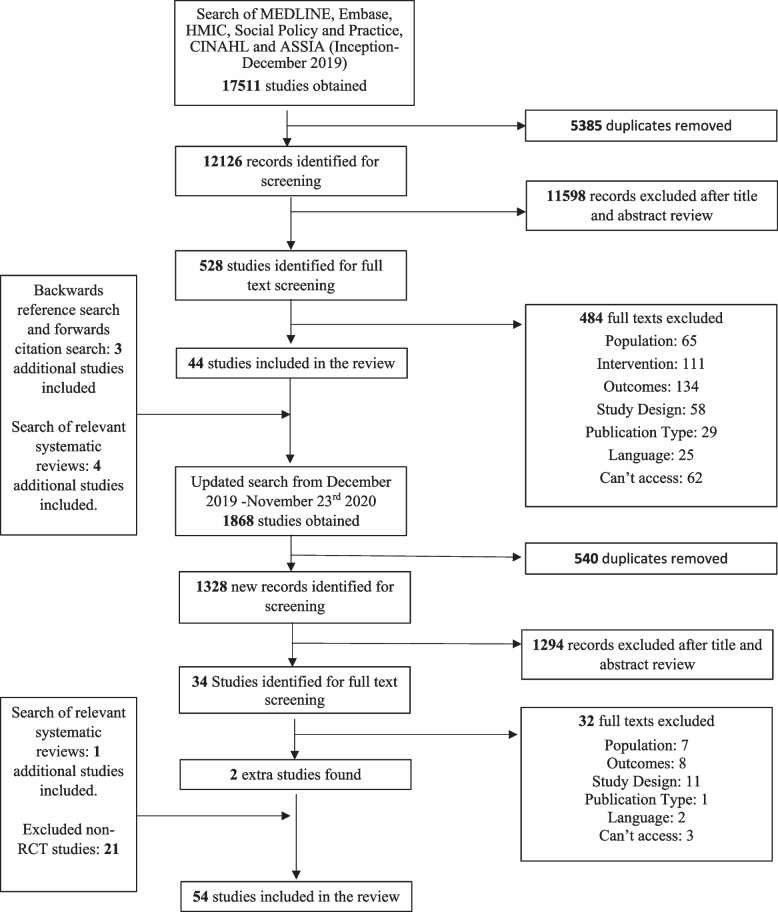
Flowchart of study selection

We excluded studies whose primary focus of treatment was not ‘personality disorder’ or associated needs. We also excluded theses and conference abstracts. Only studies published in English were included. The full search and screening process is depicted in Fig. 1.

### Study selection

After removing duplicate records, titles and abstracts were independently screened by one researcher, with 10% of them being double checked by a second researcher. The full texts of those that appeared eligible were examined independently by two researchers. Discrepancies or disagreements were resolved through discussion with the research team.

### Data extraction and quality assessment

Data were extracted by the same reviewers using a custom extraction form on Microsoft Excel and were double checked for accuracy. The standardised extraction form included study characteristics (first author name, title, year of publication, country, setting), intervention details (control category, type of treatment, treatment intensity, treatment duration), patients characteristics (number of participants, diagnostic tool), and outcome details (type of outcome, measure, measurement timepoints, effect sizes). Regarding the outcomes, in cases where more than one measures was used to assess a specific outcome (e.g., ‘BPD’ symptom severity) one of them was retained and analysed, based on the validity and frequency of use in other relevant studies. For the selection of the outcomes of interest in this meta-analysis, we consulted experts in the field (e.g., SP).

The quality of individual RCTs was assessed using the Cochrane risk of bias tool [45]. This tool provides a framework for considering risk of bias in the findings of any type of randomized trial and is structured into six domains through which bias might be introduced into the result (randomization, selection, performance, detection, attrition, reporting). Quality assessments of 10% of the studies were checked for accuracy and correct application of the tool. Any uncertainty about ratings was resolved through discussion with a senior researcher.

### Data synthesis and analysis

For the meta-analysis, effect sizes statistics were calculated as standardized mean difference (SMD), using the metafor package of the R software [46]. The latter automatically corrects the positive bias in the SMD, providing Hedges g [47]. Hedges g pools variances and standardizes outcomes across studies which allow for comparison among disparate outcome measures. Calculations used a random-effects model, which assumes that analyzed studies represent a random sample of effect sizes, facilitating generalizability [48]. Given that the studies included used different populations with differing inclusion specifications, this statistical model was considered appropriate. For the assessment of Heterogeneity, *I*^2^ statistic was calculated. A value of 0% tentatively indicates the absence of heterogeneity, and 25, 50%, or 75% signifies low, moderate, or high heterogeneity between studies, respectively [49].

Data for each of the outcomes, namely ‘personality disorder’ symptoms, ‘BPD’ symptoms, anxiety symptoms, depression symptoms, and psychiatric symptoms were analyzed in separate analyses. Active and non-active (TAU, WL, or no intervention) controls were also analyzed separately. Outcomes were grouped into categories according to the time point post-intervention they were measured: End of treatment (EOT), 1–7 months, 7–12 months, 13–18 months, and over 18 months follow-up. Subgroup analyses were also conducted to compare different intervention types for the outcomes above. Studies investigating interventions that could not be classified in any of the major intervention categories were assessed individually in a narrative synthesis. Analyses were performed on any category with at least *K* = 2 interventions. Where studies did not report outcomes at EOT but provided a follow-up of 1 month or less from EOT, this was examined as the EOT measure. A *p* value of < 0.05 was considered to be statistically significant and the following conventional values of effect size for SMD were used [50]: an effect size of 0.2 signifies a small, 0.5 a moderate, and 0.8 a large effect.

We conducted meta-regression analysis to examine the potential moderating effect of the following variables on the effectiveness of the interventions: intervention category (CBT, psychodynamic treatments, MBT, DBT, Schema Therapy), service setting (specialist day service, specialist team, standalone outpatient intervention, generic mental health service), diagnoses (‘BPD’, Other ‘personality disorders – studies assessing either a non-BPD ‘personality disorder’ or more than 1 ‘personality disorder’, CEN symptoms without a diagnosis of ‘personality disorder’ (e.g., self-harm or complex trauma), intervention intensity (< 2 sessions, 2–3 sessions, > 3 sessions per week) and intervention duration (< 4 months, 4–6 months, 7–12 months, > 12 months). Given that only a limited number of studies included participants with a diagnosis of ‘personality disorder(s)’ other than ‘BPD’, those were grouped together and were compared to studies with BPD-only sample. Meta-regressions were conducted where at least *K* = 10 studies examined the outcome for a specific timepoint [51]. As such, meta-regressions were performed for each moderator for ‘BPD’ symptoms, anxiety symptoms, depressive symptoms, and psychiatric symptoms at EOT. The degree of publication bias for each outcome was assessed by visual examination of the funnel plot and by conducting Egger’s regression test [52].

## Results

After our initial search (inception-December 2019), 17,511 studies were obtained, and 12,126 records were identified for screening. From there, we identified 526 potentially eligible studies for full text screening. After excluding 482 studies, 44 studies were included. A further three studies were found from backwards reference search and forwards citation search and four were detected from existing relevant systematic reviews. In a second updated search that aimed to identify studies from December 2019 to November 23rd, 2020, 1868 studies were obtained, and 1328 new records were identified for screening. Of those, 34 potentially eligible studies were identified for full-text screening. Two studies met the eligibility criteria and were added to the review. After searching relevant systematic reviews, one further study was included. Overall, 54 RCTs examining community treatments for ‘personality disorder’ were included in this review, and 57 comparisons between intervention and control group were meta-analysed [53–106].

### Study characteristics

The characteristics of the included studies are summarized in Table 1. In total, this review included 3716 participants, who received any type of community treatment for personality disorder. CBT was the most common intervention investigated, examined in 14 trials. Eleven trials examined psychodynamic therapies, nine examined DBT, eight assessed MBT, two investigated Schema Therapy, and 13 studies could not be grouped in any of the aforementioned intervention types and were collectively categorized as ‘Other Treatments’. The latter treatment category included either less prominent types of psychotherapy that could not be categorized in the treatment types studied (e.g., TFP), or treatments focusing on social or global outcomes, such as Nidotherapy. Forty-two studies included a non-active comparison control group, while 12 studies compared the interventions to an active control group, which received a specialist intervention.

**Table 1:** Study characteristics

Study ID^†^	Region	Setting	Sample^††^	Diagnosis	Intervention	Comparison	Outcome	Measure	Timepoints	Length (months)	Intensity (per week)	Effect size EOT (Hedge’s g) ^†††^
Abbass 2008 [53]	North America	Standalone outpatient intervention	*n* = 14 *n* = 13	Personality Disorder	Intensive short-term Dynamic Psychotherapy	TAU	Psychiatric symptoms	BSI	EOT	8	1 session	Psychiatric symptoms: *EOT*: 1.00 (0.20, 1.80)
Amianto 2011 [54]	Europe	Generic mental health service	*n* = 18 *n* = 17	BPD	Sequential Brief Adlerian Psychodynamic Psychotherapy	TAU	Psychiatric symptoms	CGI	EOT 6-month FU 12-month FU	12	1 session	Psychiatric symptoms: *EOT*: 0 (−0.68, 0.68) *6-month*: − 0.05 (− 0.73, 0.63) *12-month*: − 0.31 (− 0.99, 0.38)
Andreoli 2016 [55]	Europe	Specialist team	*n* = 140 *n* = 30	BPD	Abandonment Psychotherapy	TAU	Depressive Symptoms Psychiatric symptoms	HRSD CGI	EOT	3	2 sessions	Depressive symptoms: *EOT*: 0.59 (0.19, 0.99) Psychiatric symptoms: *EOT*: 0.81 (0.41, 1.22)
Arnevik 2009 /Antonsen 2014 [56, 57]	Europe	Specialist day service	*n* = 59 *n* = 54	Personality Disorder	Step-down Treatment	Active	Depressive symptoms Psychiatric symptoms PD Symptoms	BDI SCL-90 SCID-II	8-month FU 18-month FU 36-month FU 72-month FU	4.5	3–4 sessions	Depressive symptoms: *8-month*: 0.21 (−0.18, 0.60) *18-month*: 0.01 (− 0.32, 0.51) *36-month*: − 0.23 (− 0.67, 0,21) *72-month*: 0.04 (− 0.40, 0.48) Psychiatric symptoms: *8-month*: 0 (− 0.39, 0.39) *18-month*: − 0.12 (− 0.53, 0.29) *36-month*: − 0.17 (− 0.60, 0.27) *72-month*: 0.28 (− 0.16, 0.73) PD symptoms: *36-month*: − 0.41 (− 0.85, 0.03) *72-month*: − 0.05 (− 0.49, 0.39)
Bamelis 2014 [58]	Europe	Standalone outpatient intervention	*n* = 147 *n* = 135	Personality Disorder	Schema Therapy	TAU	PD Symptoms	SCID-II	12-month FU	12	1 session	PD symptoms: *12-month*: 0.37 (0.14, 0.61)
Bateman 1999 [59]	Europe	Specialist day service	*n* = 19 *n* = 19	BPD	MBT	TAU	Anxiety symptoms Depressive Symptoms Psychiatric symptoms	STAI BDI SCL-90	EOT	18	6 sessions (5 days)	Anxiety symptoms: *EOT*: 1.26 (0.56, 1.95) Depressive symptoms: *EOT*: 1.98 (1.21, 2.76) Psychiatric symptoms: *EOT*: 0.39 (− 0.26, 1.03)
Bateman 2001/ Bateman 2008 [60, 61]	Europe	Specialist day service	*n* = 22 *n* = 22	BPD	MBT	TAU	Anxiety symptoms Depressive Symptoms Psychiatric symptoms BPD symptoms	STAI BDI SCL-90 ZAN-BPD	6-month FU 12-month FU 18-month FU 60-month FU	18	6 sessions (5 days pw)	Anxiety symptoms: *6-month*: 0.88 (0.19, 1.56) *12-month*: 1.23 (0.52, 1.94) *18-month*: 2.39 (1.53, 3.25) Depressive symptoms: *6-month*: 1.28 (0.58, 1.99) *12-month*: 1.16 (0.47, 1.86) *18-month*: 1.15 (0.46, 1.84) Psychiatric symptoms: *6-month*: 1.04 (0.33, 1.74) *12-month*: 1.63 (0.87, 2.39) *18-month*: 2.09 (1.27, 2.91) BPD symptoms: *60-month*: 1.79 (1.02, 2.57)
Bateman 2009 [62]	Europe	Specialist team	*n* = 71 *n* = 63	BPD	MBT	Active	Depressive Symptoms Psychiatric symptoms	BDI SCL-90	EOT	18	2 sessions (combined)	Depressive symptoms: *EOT*: 0.45 (0.10, 0.79) Psychiatric symptoms: *EOT*: 0.67 (0.33, 1.02)
Berthoud 2017 [63]	Europe	Standalone outpatient intervention	*n* = 25 *n* = 25	BPD	Motive-Oriented Therapeutic Relationship	Active	Psychiatric symptoms BPD symptoms	OQ-45 BSL-23	EOT	2.5	1 session	Psychiatric symptoms: *EOT*: 0.53 (−0.08, 1.13) BPD symptoms: *EOT*: 0.01 (− 0.66, 0.68)
Blum 2008 [64]	North America	Standalone outpatient intervention	*n* = 65 *n* = 59	BPD	STEPPS	TAU	Depressive Symptoms Psychiatric symptoms BPD symptoms	BDI SCL-90/ CGI ZAN-BPD/ BEST	EOT 12-month FU	5	2-hour weekly session	Depressive symptoms: *EOT*: 0.29 (−0.16, 0.73) *12-month*: − 0.03 (− 0.47, 0.41) Psychiatric symptoms: *EOT*: 0.36 (− 0.09, 0.81) *12-month*: 0.38 (− 0.06, 0.83) BPD symptoms: *EOT*: 0.54 (0.09, 1.00) *12-month*: − 0.03 (− 0.48, 0.41)
Borschmann 2013 [65]	Europe	Generic mental health service	*n* = 46 *n* = 42	BPD	Joint Crisis Plans	TAU	Anxiety symptoms Depressive Symptoms	HADS-A HADS-D	6-month FU	1 session	1 session	Anxiety symptoms: *6 month*: − 0.38 (− 0.85, 0.08) Depressive Symptoms: *6-month*: 0.06 (− 0.41, 0.53)
Bos 2010 [66]	Europe	Standalone outpatient intervention	*n* = 42 *n* = 37	BPD	STEPPS	TAU	Psychiatric symptoms BPD symptoms	SCL-90 BPD-40	EOT 6-month FU	6	1 session	Psychiatric symptoms: *EOT*: 0.60 (0.04, 1.16) *6-month*: 0.33 (− 0.18, 0.84) BPD symptoms: *EOT*: 0.55 (0, 1.11) *6-month*: 0.34 (− 0.17, 0.84)
Bos 2011 [67]	Europe	Standalone outpatient intervention	*n* = 84 *n* = 84	BPD	STEPPS	TAU	Psychiatric symptoms BPD symptoms	SCL-90 BPD Checklist	EOT 6-month FU	6	1 session	Psychiatric symptoms: *EOT*: 0.63 (0.25, 1.01) *6-month*: 0.39 (0.02, 0.75) BPD symptoms: *EOT*: 0.34 (− 0.03, 0.71) *6-month*: 0.23 (− 0.13, 0.60)
Clarke 2013 [68]	Europe	Specialist team	*n* = 38 *n* = 40	Personality Disorder	CAT	TAU	Psychiatric symptoms	SCL-90	EOT	10	1 session	Psychiatric symptoms: *ΕΟΤ*: 0.84 (0.17, 1.50)
Clarke 2014 [69]	Europe	Specialist team	*n* = 30 *n* = 31	Personality Disorder	ACT	TAU	Depressive Symptoms Psychiatric symptoms	BDI-II SCL-90	EOT 6-month FU	4	1 session (2 hours)	Depressive symptoms: *EOT*: 0.16 (−0.48, 0.81) *6-month*: 0.46 (− 0.19, 1.11) Psychiatric symptoms: *EOT*: 0.24 (− 0.39, 0.86) *6-month*: 0.35 (− 0.28, 0.98)
Cottraux 2009 [70]	Europe	Standalone outpatient intervention	*n* = 33 *n* = 32	BPD	Cognitive Therapy	Active	Anxiety symptoms Depressive Symptoms Psychiatric symptoms	BAI BDI CGI	EOT 12-month FU	12	1 session	Anxiety symptoms: *EOT*: 0.51 (−0.13, 1.16) *12-month*: 0.94 (0.04, 1.85) Depressive symptoms: *EOT*: − 0.10 (− 0.73, 0.54) *12-month*: 0.74 (− 0.15, 1.62) Psychiatric symptoms: *EOT*: 0.30 (− 0.34, 0.94)
												*12-month*: 0.76 (− 0.13, 1.65)
Davidson 2006 /2010 [71, 72]	Europe	Generic mental health service	*n* = 54 *n* = 52	BPD	CBT	TAU	Anxiety symptoms Depressive Symptoms Psychiatric symptoms PD Symptoms	STAI BDI-II BSI SCID-II	EOT 12-month FU 60-month FU	12	1 session	Anxiety symptoms: *EOT*: 0.04 (−0.35, 0.44) *12-month*: 0.23 (− 0.17, 0.62) *60-month*: 0.20 (− 0.25, 0.66) Depressive symptoms: *EOT*: 0.11 (− 0.29, 0.50) *12-month*: 0.15 (− 0.24, 0.54) *60-month*: − 0.02 (− 0.47, 0.44) Psychiatric symptoms: *EOT*: 0.03 (− 0.36, 0.43) *12-month*: 0.12 (− 0.27, 0.51) *60-month*: − 0.07 (− 0.52, 0.39) PD symptoms: *60-month*: 0.09 (− 0.41, 0.60)
De Saeger 2014 [73]	Europe	Specialist team	*n* = 37 *n* = 37	Personality Disorder	Therapeutic assessment	Active	Psychiatric symptoms	BSI	EOT	1 session	1 session	Psychiatric Symptoms: *EOT*: − 0.12 (− 0.58, 0.33)
Doering 2010 [74]	Europe	Standalone outpatient intervention	*n* = 52 *n* = 52	BPD	Transference-Focused Psychotherapy	TAU	Anxiety symptoms Depressive Symptoms Psychiatric symptoms BPD symptoms	STAI BDI BSI No of DSM-IV criteria	EOT	12	2 sessions	Anxiety symptoms: *EOT*: − 0.15 (− 0.53, 0.24) Depressive Symptoms: *EOT*: − 0.12 (− 0.51, 0.26) Psychiatric Symptoms: *EOT*: − 0.08 (− 0.46, 0.31) BPD symptoms: *EOT*: 0.55 (0.16, 0.95)
Emmelkamp 2006 [75]	Europe	Standalone outpatient intervention	*n* = 21 *n* = 8	Avoidant Personality Disorder	CBT	Waiting List	Anxiety symptoms	LWASQ	EOT	6	1 session	Anxiety symptoms: *EOT*: − 0.02 (− 0.85, 0.81)
		Standalone outpatient intervention	*n* = 23 *n* = 8	Avoidant Personality Disorder	Brief Dynamic Therapy	Waiting List	Anxiety symptoms	LWASQ	EOT	6	1 session	Anxiety symptoms: *EOT*: −0.19 (−1.00, 0.62)
Evans 1999 [76]	Europe	Standalone outpatient intervention	*n* = 18 *n* = 16	BPD	Manual-assisted CBT	TAU	Anxiety symptoms Depressive Symptoms	HADS-A HADS-D	EOT	6	1 session	Anxiety symptoms: *EOT*: 0.62 (−0.09, 1.34) Depressive Symptoms: *EOT*: 0.87 (0.14, 1.60)
Farrell 2009 [77]	North America	Standalone outpatient intervention	*n* = 16 *n* = 16	BPD	Schema Therapy	TAU	Psychiatric symptoms BPD symptoms	SCL-90 Borderline Syndrome Index	EOT 6-month FU	8	1 session	Psychiatric symptoms: *EOT*: 1.06 (0.26, 1.86) *6-month*: 1.60 (0.74, 2.46) BPD symptoms: *EOT*: 1.66 (0.79, 2.53) *6-month*: 2.24 (1.29, 3.19)
Feigenbaum 2012 [78]	Europe	Specialist team	*n* = 26 *n* = 16	Personality Disorder	DBT	TAU	Depressive symptoms Psychiatric symptoms	BDI-II CORE-OM	EOT	12	3.5 hours	Depressive symptoms: *EOT*: −0.31 (−0.94, 0.33) Psychiatric symptoms: *EOT*: − 0.17 (− 0.80, 0.46)
Gratz 2006 [79]	North America	Standalone outpatient intervention	*n* = 12 *n* = 10	BPD	Emotion Regulation Group Intervention	TAU	Anxiety symptoms Depressive symptoms BPD symptoms	DAS-A DAS-D BEST	EOT	4	1 session	Anxiety symptoms: *EOT*: 0.89 (0.02, 1.77) Depressive symptoms: *EOT*: 1.20 (0.29, 2.11) BPD symptoms: *EOT*: 1.02 (0.12, 1.91)
Gratz 2014 [80]	North America	Standalone outpatient intervention	*n* = 31 *n* = 30	BPD	Emotion Regulation Group Intervention	TAU	Anxiety symptoms Depressive symptoms BPD symptoms	DAS-A BDI-II ZAN-BPD	EOT	3.5	1 session	Anxiety symptoms: *EOT*: 1.73 (1.14, 2.31) Depressive symptoms: *EOT*: 1.06 (0.52, 1.59) BPD symptoms: *EOT*: 1.88 (1.27, 2.48)
Gregory 2008/ 2010 [81, 82]	North America	Standalone outpatient intervention	*n* = 15 *n* = 15	BPD	Dynamic Deconstructive Psychotherapy	TAU	Depressive symptoms BPD symptoms	BDI BEST	EOT 18-month FU	12	1 session	Depressive symptoms: *EOT*: 0.50 (−0.42, 1.41) *18-month*: 0.67 (− 0.34, 1.67) BPD symptoms: *EOT*: 0.43 (− 0.49, 1.34)
												*18-month*: 0.57 (− 0.43, 1.57)
Haeyen 2018 [83]	Europe	Standalone outpatient intervention	*n* = 38 *n* = 36	Personality Disorder	Art Therapy	Waiting List	Psychiatric symptoms	OQ-45	EOT 5-week FU	2.5	1 session	Psychiatric symptoms: *EOT*: 1.23 (0.66, 1.79) *5-week*: 1.35 (0.77, 1.92)
Harned 2014 [84]	North America	Standalone outpatient intervention	*n* = 17 *n* = 9	BPD	DBT with DBT Prolonged Exposure	Active	Anxiety symptoms Depressive Symptoms Psychiatric symptoms	HAM-A HRSD BSI	ΕΟΤ 3-month FU	12	5 hours pw	Anxiety symptoms: *EOT*: 0.93 (−0.37, 2.24) *3-month*: 0.38 (− 0.87, 1.63) Depressive symptoms: *EOT*: 1.00 (− 0.32, 2.31) *3-month*: 1.07 (− 0.26, 2.39) Psychiatric symptoms: *EOT*: 1.40 (0.01, 2.78) *3-month*: 0.60 (− 0.67, 1.87)
Hellerstein 1998 [85]	North America	Standalone outpatient intervention	*n* = 25 *n* = 24	Personality Disorder	Short-Term Dynamic Psychotherapy	Active	Psychiatric symptoms	SCL-90	EOT 6-month FU	10	1 session	Psychiatric symptoms *EOT*: −0.54 (−1.32, 0.25) *6-month*: − 0.37 (−1.19, 0.44)
Jorgensen 2013/ 2014 [86, 87]	Europe	Standalone outpatient intervention	*n* = 74 *n* = 37	BPD	MBT	Active	Anxiety symptoms Depressive Symptoms Psychiatric symptoms BPD symptoms PD Symptoms	STAI BDI SCL-90 SCID-BPD SCID-II	EOT 6-month FU 18-month FU	24	2 sessions	Anxiety symptoms: *EOT*: 0.24 (− 0.26, 0.75) *6-month*: 0.69 (0.08, 1.30) *18-month*: 0.58 (− 0.01, 1.17) Depressive symptoms: *EOT*: 0.32 (− 0.18, 0.82) *6-month*: 0.38 (− 0.22, 0.98) *18-month*: 0.33 (− 0.26, 0.91) Psychiatric symptoms: *EOT*: 0.25 (− 0.26, 0.75) *6-month*: 0.27 (− 0.33, 0.87) *18-month*: 0.39 (− 0.20, 0.97) BPD symptoms: *EOT*: 0.33 (− 0.17, 0.84) *18-month*: 0.12 (− 0.46, 0.70)
												PD symptoms: *18-month*: − 0.08 (− 0.89, 0.73)
Khabir 2018 [88]	Asia	Standalone outpatient intervention	*n* = 12 *n* = 6	BPD	MBT	No intervention	BPD symptoms	BPDSI-IV	EOT 2-month FU	3	2 sessions	BPD symptoms: *EOT*: 1.33 (0.26, 2.41) *2-month*: 2.05 (0.86, 3.24)
		Standalone outpatient intervention	*n* = 12 *n* = 6	BPD	DBT	No intervention	BPD symptoms	BPDSI-IV	EOT 2-month FU	3	2 sessions	BPD symptoms: *EOT*: 1.69 (0.57, 2.82) 2-month: 2.22 (1.00, 3.44)
Koons 2001 [89]	North America	Specialist day services	*n* = 14 *n* = 14	BPD	DBT	TAU	Anxiety symptoms Depressive Symptoms, BPD symptoms	HAM-A BDI No of DSM-IV criteria	EOT	6	2.5 hours	Anxiety symptoms: *EOT*: 1.22 (0.27, 2.18) Depressive symptoms: *EOT*: 1.12 (0.18, 2.06) BPD symptoms: *EOT*: 0.29, (−0.59, 1.17)
Kramer 2014 [90]	Europe	Standalone outpatient intervention	*n* = 42 *n* = 43	BPD	Motive-Oriented Therapeutic Relationship	Active	Psychiatric symptoms BPD symptoms	OQ-45 BSL-23	EOT	3	1 session	Psychiatric symptoms: *EOT*: −0.14, (−0.60, 0.31) BPD symptoms: *EOT*: − 0.07 (− 0.53, 0.39)
Kramer 2016 [91]	Europe	Standalone outpatient intervention	*n* = 21 *n* = 20	BPD	DBT	TAU	Psychiatric symptoms	OQ-45	EOT	5	1 session	Psychiatric symptoms: *EOT*: 0.35 (−0.26, 0.97)
Laurenssen 2018 [92]	Europe	Specialist day service	*n* = 54 *n* = 51	BPD	Day Hospital MBT	Active	Depressive Symptoms Psychiatric symptoms BPD symptoms	BDI BSI BPDSI-IV	EOT	18	5 hours, 5 days	Depressive symptoms: *EOT*: −0.10 (−0.51, 0.31) Psychiatric symptoms: *EOT*: − 0.18 (− 0.58, 0.23) BPD symptoms: *EOT*: 0.07 (− 0.34, 0.47)
Leppanen 2016 [93]	Europe	Standalone outpatient intervention	*n* = 24 *n* = 47	BPD	Community Treatment by Experts	TAU	BPD symptoms	BPDSI-IV	EOT	12	1 session	BPD symptoms: *EOT*: 0.35 (−0.22, 0.93)
McMain 2012 [94]	North America	Standalone outpatient intervention	*n* = 90 *n* = 90	BPD	DBT	Active	Depressive Symptoms Psychiatric symptoms BPD symptoms	BDI-II SCL-90 ZAN-BPD	EOT 6-month FU 12-month FU 18-month FU 24-month FU	12	3.5 hours	Depressive symptoms: *EOT*: 0.17 (−0.12, 0.47) *6-month*: 0.13 (− 0.16, 0.42) *12-month*: − 0.04 (− 0.33, 0.26) *18-month*: − 0.09 (− 0.38, 0.20) *24-month*: − 0.39 (− 0.68, − 0.09) Psychiatric symptoms: EOT: 0.02 (− 0.27, 0.32) *6-month*: 0.09 (− 0.20, 0.38) *12-month*: 0 (− 0.29, 0.29) *18-month*: − 0.10 (− 0.39, 0.20) *24-month*: − 0.26 (− 0.55, 0.03) BPD symptoms: *EOT*: 0.04 (− 0.25, 0.33) *6-month*: 0.25 (− 0.05, 0.54) *12-month*: − 0.01 (− 0.31, 0.28) *18-month*: 0.19 (− 0.11, 0.48) *24-month*: − 0.27 (− 0.57, 0.02)
McMain 2017 [95]	North America	Standalone outpatient intervention	*n* = 42 *n* = 42	BPD	Brief DBT Skills Training	Waiting List	Depressive Symptoms Psychiatric symptoms BPD symptoms	BDI SCL-90 BSL-23	EOT 3-month FU	5	1 session	Depressive symptoms: *EOT*: 0.53 (0.09, 0.97) *3-month*: 0.10 (− 0.33, 0.53) Psychiatric symptoms: *EOT*: 0.76 (0.31, 1.20) *3-month*: 0.27 (− 0.16, 0.70) BPD symptoms: *EOT*: 0.71 (0.27, 1.15) *3-month*: 0.20 (− 0.23, 0.63)
Morton 2012 [96]	Oceania	Specialist team	*n* = 21 *n* = 20	BPD	ACT Group Treatment	TAU	Anxiety symptoms Depressive Symptoms BPD symptoms	DASS-A DASS-D BEST	EOT	3	1 session (2 hours)	Anxiety symptoms: *EOT*: 0.66 (0.03, 1.29) Depressive symptoms: *EOT*: 0.67 (0.05, 1.30) BPD symptoms: *EOT*: 1.22 (0.55, 1.89)
Pascual 2015 [97]	Europe	Standalone outpatient intervention	*n* = 36 *n* = 34	BPD	Cognitive Rehabilitation	Active	Anxiety symptoms Depressive Symptoms BPD symptoms	HAM-A MADRS BSL-23	EOT 6-month FU	4	1 session (2 hours)	Anxiety symptoms: *EOT*: − 0.09 (− 0.67, 0.49) *6-month*: − 0.39 (−1.00, 0.22) Depressive symptoms: *EOT*: − 0.29 (− 0.87, 0.29) *6-month*: − 1.05 (− 1.69, − 0.40) BPD symptoms: *EOT*: 0.15 (− 0.73, 0.43) *6-month*: 0.19 (− 0.42, 0.79)
Pearce 2017 [98]	Europe	Specialist day service	*n* = 35 *n* = 35	Personality Disorder	Democratic Therapeutic community	TAU	Psychiatric symptoms	GHQ	EOT	24	5 to 15 hours	Psychiatric symptoms: *EOT*: 0.72 (0.07, 1.38)
Popolo 2019 [99]	Europe	Generic mental health service	*n* = 10 *n* = 10	Personality Disorder	Metacognitive Interpersonal Therapy	TAU	Psychiatric symptoms	CORE-OM	EOT	4	1 session (2 hours)	Psychiatric symptoms: *EOT*: 1.06 (0.07, 2.06)
Priebe 2012 [100]	Europe	Standalone outpatient intervention	*n* = 40 *n* = 40	Personality Disorder	DBT	TAU	Psychiatric symptoms BPD symptoms	BSI ZAN-BPD	EOT	12	3.5 hours plus telephone consult.	Psychiatric symptoms: *EOT*: 0.28 (−0.23, 0.79) BPD symptoms: *EOT*: 0.38 (− 0.09, 0.86)
Ranger 2009 [101]	Europe	Generic mental health service	*n* = 28 *n* = 24	Other Diagnoses/ symptoms	Nidotherapy	TAU	Psychiatric symptoms	BPRS	EOT	12	1 session	Psychiatric symptoms: *EOT*: 0.44 (− 0.21, 1.10)
Reneses 2013 [102]	Europe	Generic mental health service	*n* = 25 *n* = 28	BPD	Psychic Representation Focused Psychotherapy	TAU	Anxiety symptoms Depressive Symptoms, Psychiatric symptoms BPD symptoms	STAI MADRS SCL-90 ZAN-BPD	EOT	6	1 session	Anxiety symptoms: *EOT*: 0.52 (−0.09, 1.13) Depressive symptoms: *EOT*: 0.56 (− 0.05, 1.17) Psychiatric symptoms: *EOT*: 0.55 (− 0.06, 1.16) BPD symptoms: *EOT*: 0.82 (0.19, 1.44)
Soler 2009 [103]	Europe	Standalone outpatient intervention	*n* = 29 *n* = 30	BPD	DBT Skills Training	TAU	Anxiety symptoms	HAM-A HRSD	EOT	3	1 session (2 hours)	Anxiety symptoms: *EOT*: 0.65 (− 0.11, 1.41)
							Depressive Symptoms, Psychiatric symptoms BPD symptoms	SCL-90 CGI-BPD				Depressive symptoms: *EOT*: 1.01 (0.23, 1.80) Psychiatric symptoms: *EOT*: 0.40 (−0.35, 1.15) BPD symptoms: *EOT*: 0.90 (0.13, 1.68)
Svartberg 2004 [104]	Europe & North America	Standalone outpatient intervention	*n* = 25 *n* = 25	BPD	Short term Dynamic Psychotherapy	TAU	Psychiatric symptoms PD Symptoms	SCL-90 MCMI	EOT 6-month FU 12-month FU 24-month FU	10	1 session	Psychiatric symptoms: *EOT*: 0.11 (−0.45, 0.66) *6-month*: 0.42 (− 0.16, 1.00) *12-month*: 0.19 (− 0.39, 0.77) *24-month*: 0.10 (− 0.49, 0.69) PD symptoms: *EOT*: 0.25 (− 0.32, 0.81) *24-month*: 0.17 (− 0.40, 0.73)
Vinnars 2005 [105]	Europe	Standalone outpatient intervention	*n* = 76 *n* = 80	Personality Disorder	Psychodynamic Therapy	TAU	Psychiatric symptoms PD Symptoms	SCL-90 No of DSM-IV Axis II criteria	EOT 12-month FU	12	1 session	Psychiatric symptoms: *EOT*: 0.18 (−0.20, 0.55) *12-month*: 0.14 (− 0.21, 0.49) PD symptoms: *EOT*: 0.20 (− 0.17, 0.57) *12-month*: 0.07 (− 0.29, 0.42)
Winston 1994 [106]	North America	Standalone outpatient intervention	*n* = 30 *n* = 13	Personality Disorder	Brief Adaptive Psychotherapy	Waiting List	Psychiatric symptoms	SCL-90	EOT	10	1 session	Psychiatric symptoms: *EOT*: 1.27 (0.56, 1.97)
	North America	Standalone outpatient intervention	*n* = 25 *n* = 13	Personality Disorder	Short-term Dynamic Psychotherapy	Waiting List	Psychiatric symptoms	SCL-90	EOT	10	1 session	Psychiatric symptoms: *EOT*: 0.71 (0.02, 1.40)

^†^Studies containing follow-up data were merged with the original studies. ^††^Number of participants allocated to each group. The first row reports the number of participants included in the experimental (intervention) group, while the second row included the number of participants allocated to the control group. ^†††^Effect sizes of each study were transformed to Hedges’ g for the purposes of the meta-analysis and are reported as such with their corresponding 95% CIs. Abbreviations: *EOT* End of Treatment, *FU* Follow-up, *BPD* Borderline Personality Disorder, *PD* Personality Disorder, *TAU* Treatment as Usual, *DBT* Dialectical Behavior Therapy, *ACT* Acceptance and Commitment Therapy, *ΜΒΤ* Mentalization-Based Treatment, *CBT* Cognitive Behavioural Therapy, *CAT* Cognitive Analytic Therapy, *STEPPS* Systems Training for Emotional Predictability and Problem Solving, *BSI* Brief Symptom Inventory, *CGI* Clinical Global Impression, *HRSD* Hamilton Rating Scale for Depression, *BDI* Beck’s Depression Inventory, *SCL-90* Symptom Checklist 90, *SCID-II* Structured Clinical Interview for DSM-IV Axis II Disorders, *STAI* State-Trait Anxiety Inventory, *ZAN-BPD* Zanarini Rating Scale for Borderline Personality Disorder, *MCMI* Millon Clinical Multiaxial Inventory, *HAM-A* Hamilton Anxiety Rating Scale, *MADRS* Montgomery-Åsberg Depression Rating Scale, *BPRS* Brief Psychiatric Rating Scale, *CORE-OM* Clinical Outcomes in Routine Evaluation-Outcome Measure, *GHQ* General Health Questionnaire, *BSL-23* Borderline Symptom List, *BEST* Borderline Evaluation of Severity over Time, *DASS-(A/D)* Depression Anxiety Stress Scales (Anxiety/ Depression), *BPDSI* Borderline Personality Disorder Severity index, *OQ-45* Outcome Questionnaire 45, *LWASQ* Lehrer Woolfolk Anxiety Symptom Questionnaire, *HADS-(A/D)* Hospital Anxiety and Depression Scale (Anxiety/ Depression)

Thirty-two studies included interventions delivered as standalone outpatient interventions, seven studies included interventions that were provided in specialist day services, in seven studies interventions were offered in generic mental health services, and in seven studies interventions were delivered by a specialist team. Thirty-seven studies included participants diagnosed with ‘BPD’, while 16 studies included participants diagnosed with a specific ‘non-BPD’ ‘personality disorder’ (e.g. ‘avoidant personality disorder’) or a sample of participants diagnosed with different ‘personality disorders’. One study included participants with either a diagnosis of ‘personality disorder’ or presenting personality difficulties without a formal diagnosis. Most studies (*K* = 38) were conducted in European countries, 13 were conducted in North America, one in Australia, one in Iran, and one included two sites, one in Norway and one in Canada.

### Quality assessment and publication bias

Overall, the risk of bias examination indicated that the quality of the studies was low to moderate, although large variations were observed between studies (Additional file 2, Fig. S1). The majority of the studies reported adequate random sequence allocation, while fewer reported adequate allocation concealment. All included studies were assessed as having high performance bias, something common in complex psychosocial interventions, where therapists and participants cannot be blinded. More than half of the studies displayed high risk of detection bias, as most of the outcomes were measured using self-report measures. Around one quarter of the studies referred to a protocol that could be found online, while the rest did not report having one. More than three quarters of the studies were assessed as having low risk of other bias, that would emerge from broader methodological errors or omissions.

The funnel plots (Additional file 3, Fig. S2) showed that there is considerable asymmetry for ‘BPD’ symptoms, depressive symptoms, and psychiatric symptoms, indicating publication bias. The results from the Egger’s test confirmed the absence of publication bias for anxiety symptoms (*p* = 0.12) and the presence of publication bias for ‘BPD’ symptoms (*p* = 0.02), depressive symptoms (*p* = 0.02), and psychiatric symptoms (*p* = 0.05).

### ‘Personality disorder’ symptoms

Two studies assessing general personality disorder symptoms at > 18 months follow-up found weak evidence in favor of the interventions compared to TAU/WL (*g* = 0.25, 95% CI: − 0.05 to 0.55, *p* = 0.10, *I*^2^ = 50.49%), without reaching statistical significance. Three studies found no difference in personality disorder symptoms between the interventions under investigation and the active control interventions at > 18 months follow-up (*g* = − 0.13, 95% CI: − 0.45 to 0.19, *p* = 0.43, *I*^2^ = 26.83%).

### ‘BPD’ symptoms

**Fig. 2:**
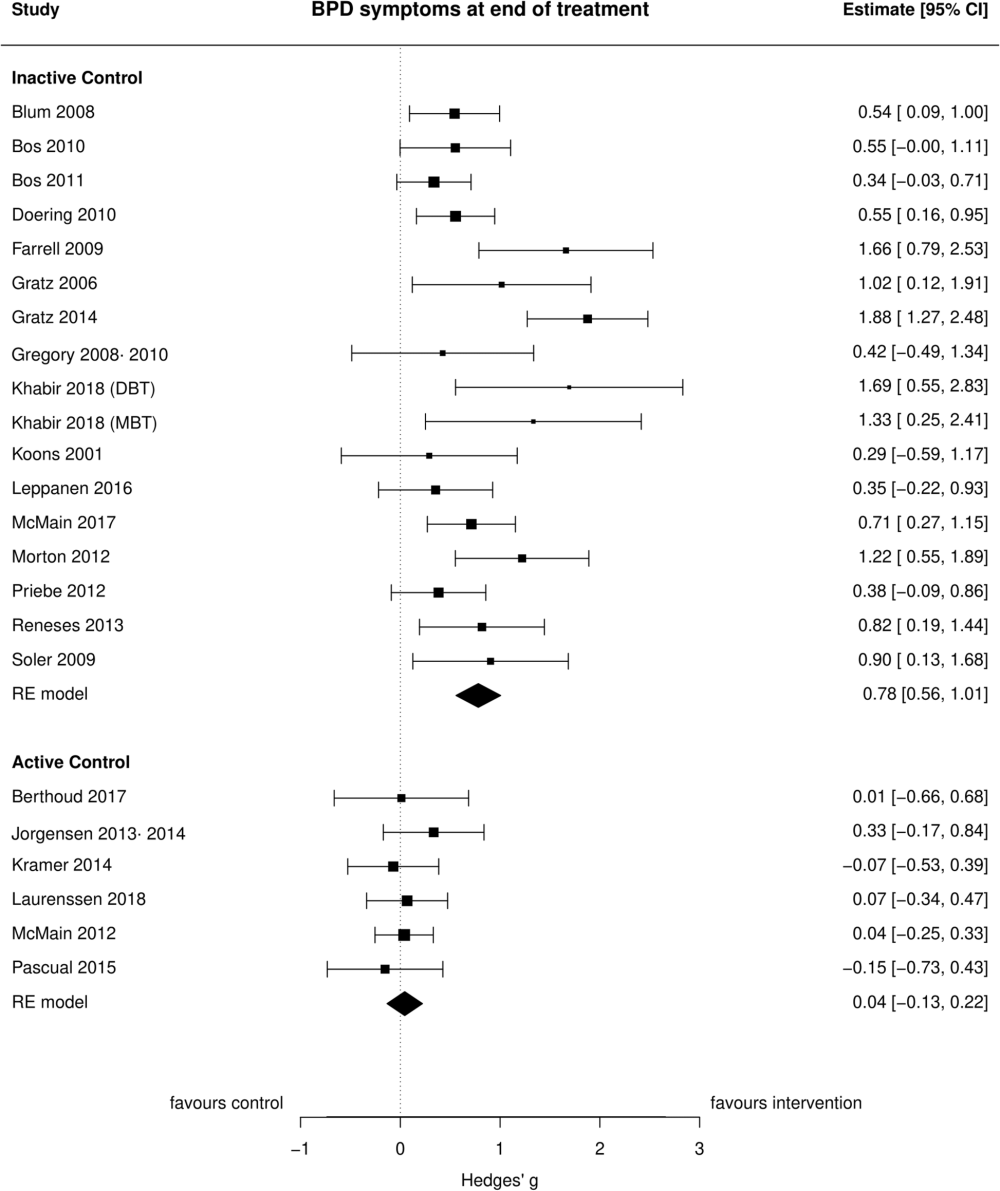
Forest plot of BPD symptoms at end of treatment

In total 23 comparisons for ‘BPD’ symptoms at EOT were meta-analyzed. Seventeen comparisons comparing interventions to TAU/WL yielded a large pooled effect size of *g* = 0.78 (95% CI: 0.56 to 1.01, *p* < 0.0001, *I*^2^ = 55.40%), providing strong evidence in favor of the interventions for this outcome (Fig. 2). CBT (*K* = 6) and psychodynamic therapies (*K* = 2) significantly reduced ‘BPD’ symptom severity compared to TAU/WL (*g* = 0.89, 95% CI: 0.42 to 1.36, *p* = 0.0002, *I*^2^ = 76.85% and *g* = 0.69, 95% CI: 0.18 to 1.21, *p* = 0.0085, *I*^2^ = 0.00%, respectively), suggesting strong evidence for the efficacy of both intervention types. Studies assessing DBT for ‘BPD’ symptoms (*K* = 5) found that this intervention significantly reduced ‘BPD’ symptom severity (*g* = 0.67, 95% CI: 0.32 to 1.01, *p* = 0.0002, *I*^2^ = 28.07%), compared to TAU/WL. Six studies comparing the interventions under investigation and the active control interventions found that both treatments were equally effective in reducing ‘BPD’ symptom severity at EOT (*g* = 0.04, 95% CI: − 0.13 to 0.22, *p* = 0.62, *I*^2^ = 0.00%).

The efficacy of the interventions compared to TAU/WL for ‘BPD’ symptoms was maintained (*K* = 6) when assessed at < 7 months follow-up (*g* = 1.01, 95% CI: 0.37 to 1.65, *p* = 0.002, *I*^2^ = 83.68%). Two studies compared the interventions and the active control interventions at < 7 months follow-up and found that there was weak evidence in favor of the interventions (*g* = 0.24, 95% CI: − 0.03 to 0.50, *p* = 0.08, *I*^2^ = 0.00%). Both the interventions under investigation and the active control interventions were equally effective at 7–12 months follow up (*K* = 2) (*g* = 0.17, 95% CI: − 0.09 to 0.43, *p* = 0.19, *I*^2^ = 0.00%).

### Anxiety symptoms

Anxiety symptoms were assessed as an outcome at EOT in 15 trials, and 16 comparisons were available. Twelve of the comparisons yielded a pooled effect size of *g* = 0.58 (95% CI: 0.21 to 0.95, *p* = 0.002, *I*^2^ = 75.29%), favoring community interventions over TAU/WL (Table 2). The pooled effect size for CBT (*K* = 6) and DBT (*K* = 2) for anxiety symptoms at EOT was *g* = 0.65 (95% CI: 0.08 to 1.23, *p* = 0.0255, *I*^2^ = 79.29%), and *g* = 0.87 (95% CI: 0.28 to 1.47, *p* = 0.004, *I*^2^ = 0.00%), respectively, indicating strong evidence for the efficacy of both interventions compared to TAU/WL. Psychodynamic treatments (*K* = 2) did not reduce anxiety symptoms at EOT compared to TAU/WL (*g* = 0.21, 95% CI: − 0.47 to 0.90, *p* = 0.54, *I*^2^ = 46.26%). Community interventions were not more effective compared to active control interventions (*K* = 4) in reducing anxiety symptom severity at EOT (*g* = 0.25, 95% CI: − 0.07 to 0.57, *p* = 0.13, *I*^2^ = 0.00%).

**Table 2:** Meta-analysis for all outcomes at all time-points

Outcome	Intervention Type	Comparison Type	Timepoint^†^	K (Number of comparisons)	Hedges’ g (95% CI)^††^	*p*	*I*^2^
Anxiety symptoms	All	TAU/WL	End of treatment	11 (12)	0.58 (0.21, 0.95)	0.002	75.29%
			7–12 months FU	2 (2)	0.68 (−0.30, 1.66)	0.17	82.83%
	CBT		End of treatment	6 (6)	0.65 (0.08, 1.23)	0.0255	79.29%
	DBT		End of treatment	2 (2)	0.87 (0.28, 1.47)	0.004	0.00%
	Psychodynamic Treatment		End of treatment	2 (2)	0.21 (−0.47, 0.90)	0.54	46.26%
	All	Active	End of treatment	4 (4)	0.25 (−0.07, 0.57)	0.13	0.00%
			< 7 months FU	4 (4)	0.01 (−0.57, 0.60)	0.96	67.55%
Depressive symptoms	All		End of Treatment	16 (16)	0.57 (0.32, 0.83)	< 0.0001	67.94%
		TAU/WL	< 7 month FU	3 (3)	0.57 (−0.11, 1.25)	0.01	74.70%
			7–12 months FU	3 (3)	0.36 (−0.22, 0.95)	0.225	76.11%
			13–18 months FU	2 (2)	0.99 (0.42, 1.57)	0.0007	0.00%
	CBT		End of Treatment	7 (7)	0.55 (0.23, 0.88)	0.0009	54.88%
	DBT		End of Treatment	4 (4)	0.53 (−0.06, 1.13)	0.08	68.39%
	Psychodynamic Treatment		End of Treatment	2 (2)	0.54 (0.03, 1.05)	0.04	0.00%
	All	Active	End of Treatment	7 (7)	0.15 (−0.07, 0.37)	0.18	33.64%
			< 7 months FU	5 (5)	0.02 (−0.46, 0.50)	0.94	72.80%
			7–12 months FU	3 (3)	0.14 (−0.17, 0.46)	0.38	35.93%
			13–18 months FU	3 (3)	0.15 (−0.17, 0.48)	0.36	0.00%
			> 18 months FU	3 (3)	−0.24 (−0.48, 0.01)	0.055	19.59%
	DBT		End of Treatment	2 (2)	0.32 (−0.30, 0.94)	0.31	28.94%
	MBT		End of Treatment	3 (3)	0.23 (−0.12, 0.57)	0.1950	51.93%
Psychiatric symptoms	All	TAU/WL	End of Treatment	24 (25)	0.50 (0.35, 0.66)	< 0.0001	48.56%
			< 7 month FU	8 (8)	0.61 (0.26, 0.95)	0.0006	67.07%
			7–12 months FU	5 (5)	0.34 (−0.10, 0.78)	0.13	75.18%
	CBT		End of Treatment	7 (7)	0.45 (0.21, 0.69)	0.0002	31.43%
	Psychodynamic Therapy		End of Treatment	4 (4)	0.50 (0.14, 0.86)	0.007	34.35%
	DBT		End of Treatment	5 (5)	0.36 (0.05, 0.67)	0.02	31.91%
	All	Active	End of Treatment	11 (11)	0.14 (−0.09, 0.37)	0.23	55.77%
			< 7 month FU	5 (5)	0.15 (−0.08, 0.37)	0.21	0.00%
			7–12 months FU	4 (4)	0.07 (−0.14, 0.28)	0.53	0.00%
			13–18 months FU	3 (3)	−0.02 (−0.27, 0.23)	0.87	14.19%
			> 18 months FU	4 (4)	−0.06 (−0.32, 0.20)	0.66	33.92%
	Psychodynamic Therapy		End of Treatment	4 (4)	0.02 (−0.37, 0.40)	0.93	42.76%
	DBT		End of Treatment	2 (2)	0.53 (−0.77, 1.83)	0.42	71.27%
	MBT		End of Treatment	3 (3)	0.26 (−0.27, 0.79)	0.34	79.42%
BPD symptoms	All	TAU/WL	End of Treatment	16 (17)	0.78 (0.56, 1.01)	< 0.0001	55.40%
			< 7 month FU	6 (6)	1.01 (0.37, 1.65)	0.002	83.68%
	CBT		End of Treatment	6 (6)	0.89 (0.42, 1.36)	0.0002	76.85%
	Psychodynamic Therapy		End of Treatment	2 (2)	0.69 (0.18, 1.21)	0.0085	0.00%
	DBT		End of Treatment	5 (5)	0.67 (0.32, 1.01)	0.0002	28.07%
	All	Active	End of Treatment	6 (6)	0.04 (−0.13, 0.22)	0.62	0.00%
			< 7 month FU	2 (2)	0.24 (−0.03, 0.50)	0.08	0.00%
			13–18 months FU	2 (2)	0.17 (−0.09, 0.43)	0.19	0.00%
	Psychodynamic Therapy		End of Treatment	2 (2)	−0.05 (−0.42, 0.33)	0.81	0.00%
	MBT		End of Treatment	2 (2)	0.17 (−0.14, 0.49)	0.28	0.00%
PD Symptoms	All	TAU/WL	7–12 months FU	2 (2)	0.25 (−0.05, 0.55)	0.1	50.49%
		Active	> 18 months FU	3 (3)	−0.13 (−0.45, 0.19)	0.43	26.83%

^†^Meta-analysis was conducted where K ≥ 2. ^††^Effect sizes were transformed to Hedges’ g for the purposes of the meta-analysis and are reported as such with their corresponding 95% confidence intervals *Abbreviations*: *ΜΒΤ* Mentalization-Based Treatment, *CBT* Cognitive Behavioral Interventions, *FU* Follow-up, *BPD* Borderline Personality Disorder, *DBT* Dialectical Behavior Therapy

Two comparisons provided a medium, but not significant effect size (*g* = 0.68, 95% CI: − 0.30 to 1.66, *p* = 0.17, *I*^2^ = 82.83%) for the effectiveness of interventions compared to TAU/WL for anxiety symptoms at 7–12 months follow-up. The pooled Hedge’s g for the effectiveness of interventions compared to active control interventions for anxiety symptoms at < 7 months follow-up (*K* = 4) was not significant (*g* = 0.01, 95% CI: − 0.57 to 0.60, *p* = 0.96, *I*^2^ = 67.55%), indicating comparable effectiveness of the two groups.

### Depressive symptoms

Overall, 23 comparisons compared interventions to control conditions for depressive symptoms at EOT. Sixteen comparisons comparing interventions to TAU/WL demonstrated a medium effect size (*g* = 0.57, 95% CI: 0.32 to 0.83, *p* = < 0.0001, *I*^2^ = 67.94%) providing strong evidence favoring interventions over TAU/WL. Seven studies provided a pooled effect size of *g* = 0.55 (95% CI: 0.23 to 0.88, *p* = 0.0009, *I*^2^ = 54.88%) for the effectiveness of CBT compared to TAU/WL, suggesting strong evidence in favor of the former over TAU/WL. Four comparisons between DBT and TAU/WL yielded an effect size of *g* = 0.53 (95% CI: − 0.06 to 1.13, *p* = 0.08, *I*^2^ = 68.39%) suggesting weak evidence of the efficacy of DBT, while two comparisons between Psychodynamic Therapy and TAU/WL provided a medium effect size of *g* = 0.54 (95% CI: 0.03 to 1.05, *p* = 0.04, *I*^2^ = 0.00%), indicating its superiority compared to TAU/WL. Seven studies comparing the interventions under investigation and the active control interventions found that the former were not superior in reducing depression symptoms at EOT (*g* = 0.15, 95% CI: − 0.07 to 0.37, *p* = 0.18, *I*^2^ = 33.64%).

A total of three comparisons between interventions and TAU/WL at < 7 months follow-up provided an effect estimate of *g* = 0.57 (95% CI: − 0.11 to 1.25, *p* = 0.10, *I*^2^ = 74.70%), which suggests weak evidence in favor of the interventions for depressive symptoms, without reaching statistical significance. The pooled effect size from three studies examining the comparisons of interventions and TAU/WL at 7–12 months follow-up was small (*g* = 0.36, 95% CI: − 0.22 to 0.95, *p* = 0.225, *I*^2^ = 76.11%) and not significant. Two comparisons between interventions and TAU/WL at 13–18 months follow-up provided a large pooled effect size of *g* = 0.99 (95% CI: 0.42 to 1.57, *p* = 0.0007, *I*^2^ = 0.00%), suggesting strong evidence for the maintenance of the effectiveness of the interventions for depressive symptoms at this follow-up point.

Five studies found that there was no difference between the interventions under investigation and their active control interventions in reducing depressive symptom severity at < 7 months follow-up (*g* = 0.02, 95% CI: − 0.46 to 0.50, *p* = 0.94, *I*^2^ = 72.80%). Non-significant difference between interventions and the active controls (*K* = 3) was found for 7–12 months follow-up (*g* = 0.14, 95% CI: − 0.17 to 0.46, *p* = 0.38, *I*^2^ = 35.93%) and for 13–18 months follow-up (*K* = 3) (*g* = 0.15, 95% CI: − 0.17 to 0.48, *p* = 0.36, *I*^2^ = 0.00%). At > 18 months follow-up, three comparisons between the interventions and the active controls found a small and marginally significant pooled effect size (*g* = − 0.24, 95% CI: − 0.48 to 0.01, *p* = 0.055, *I*^2^ = 19.59), indicating less effectiveness of the interventions compared to the active controls in reducing depression symptoms.

### Psychiatric symptoms

**Fig. 3:**
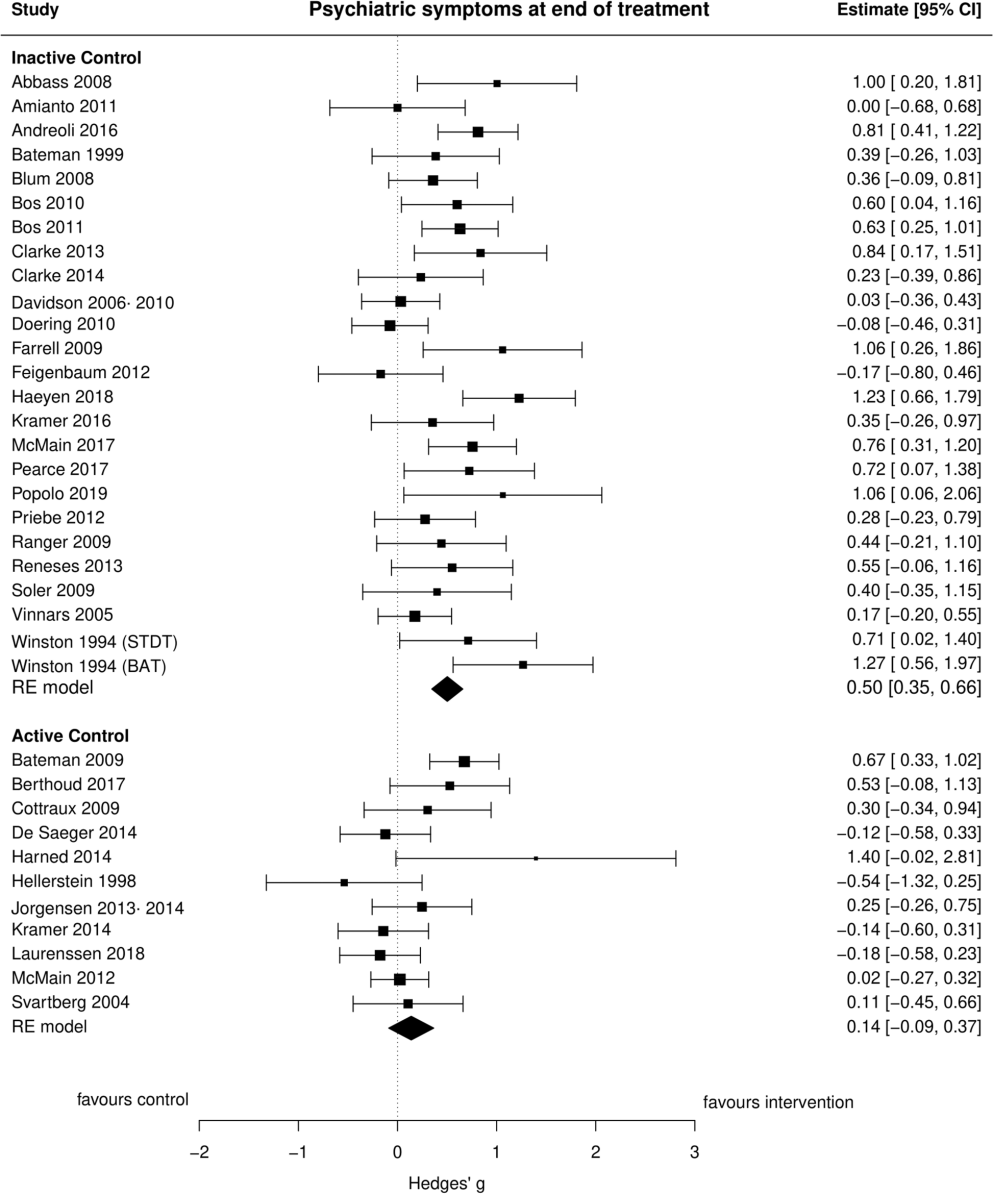
Forest plot of psychiatric symptoms at end of treatment

Overall, 36 comparisons were made between interventions and control conditions on psychiatric symptoms at EOT (Fig. 3). The pooled effect estimate from 25 comparisons comparing interventions and TAU/WL provided a moderate effect size (*g* = 0.50, 95% CI: 0.35 to 0.66, *p* < 0.0001, *I*^2^ = 48.56%), suggesting strong evidence for the efficacy of the interventions compared to TAU/WL. Seven studies reporting on psychiatric symptoms at EOT favored CBT over TAU/WL (*g* = 0.45, 95% CI: 0.21 to 0.69, *p* = 0.0002, *I*^2^ = 31.43%). Psychodynamic treatment, examined by four studies, had strong evidence of a moderate effect on reducing psychiatric symptoms (*g* = 0.50, 95% CI: 0.14 to 0.86, *p* = 0.007, *I*^2^ = 34.35) compared to TAU/WL. Evidence for the effectiveness of DBT (*g* = 0.36, 95% CI: 0.05 to 0.67, *p* = 0.02, *I*^2^ = 31.91%) compared to TAU/WL was reported by five studies on psychiatric symptoms. Eleven studies compared interventions and active control interventions and found equal efficacy for psychiatric symptoms at EOT (*g* = 0.14, 95% CI: − 0.09 to 0.37, *p* = 0.23, *I*^2^ = 55.77%).

Eight comparisons between interventions and TAU/WL that reported on psychiatric symptoms at < 7 months follow-up favored interventions over control comparisons (*g* = 0.61, 95% CI: 0.26 to 0.95, *p* = 0.0006, *I*^2^ = 67.07%) providing strong evidence for the maintenance of effectiveness at this follow-up point. The pooled effect size was smaller (*g* = 0.34, 95% CI: − 0.10 to 0.78, *p* = 0.13, *I*^2^ = 75.18%) and not significant when interventions were compared to TAU/WL (*K* = 5) at 7–12 months follow-up. Studies comparing the interventions to active control interventions at different follow-up points found that both groups were equally effective at < 7 months follow-up (*K* = 5) (*g* = 0.15, 95% CI: − 0.08 to 0.37, *p* = 0.21, *I*^2^ = 0.00%), at 7–12 months follow-up (*K* = 4) (*g* = 0.07, 95% CI: − 0.14 to 0.28, *p* = 0.53, *I*^2^ = 0.00%), at 13–18 months follow-up (*K* = 3) (*g* = − 0.02, 95% CI: − 0.27 to 0.23, *p* = 0.87, *I*^2^ = 14.19%) and at > 18 months follow-up (*K* = 4) (*g* = − 0.06, 95% CI: − 0.32 to 0.20, *p* = 0.66, *I*^2^ = 33.92%).

### Narrative synthesis

Thirteen studies were included in the Other Treatments category, and a brief narrative synthesis was conducted on the efficacy of each of the interventions compared to control comparisons.

Two interventions were aimed at the reorganization of the services for people with personality disorder. Step-down treatment was not effective in reducing personality disorder symptoms, depressive symptoms, and psychiatric symptoms compared to outpatient therapy [56, 57]. In contrast, Democratic Therapeutic Community, a form of psychosocial treatment based on a collaborative approach to staff–patient interaction, significantly reduced the severity of psychiatric symptoms (*g* = 0.72, 95% CI: 0.07 to 1.38) compared to TAU [98].

Three studies examined interventions designed to enhance self-management and provide care. In a one-session intervention study, Joint Crisis Plans did not reduce anxiety symptoms and depressive symptoms at 6 months follow-up compared to TAU [65]. Another single-session trial found that therapeutic assessment was not superior compared to goal-focused pretreatment intervention in reducing psychiatric symptom severity [73]. Finally, a trial comparing Nidotherapy and TAU for psychiatric symptom severity, found that both interventions were equally effective for this outcome [101].

Another study that compared Sequential Brief Adlerian Psychodynamic Psychotherapy plus supervised team management and supervised team management alone found no difference between the two interventions in reducing psychiatric symptoms [54]. Abandonment psychotherapy was found to reduce the severity of depressive (*g* = 0.59, 95% CI: 0.19 to 0.99) and psychiatric symptoms (*g* = 0.81, 95% CI: 0.41 to 1.22) compared to TAU [55]. In a study it was found that TFP did not reduce depressive, anxiety, and psychiatric symptoms compared to TAU, however, it could significantly reduce ‘BPD’ symptoms (*g* = 0.55, 95% CI: 0.16 to 0.95) [74]. Another trial found no difference between Community Treatment by Experts and TAU in treating ‘BPD’ symptoms [93]. Brief adaptive therapy could significantly reduce psychiatric symptoms (*g* = 1.27, 95% CI: 0.56 to 1.97) compared to WL at EOT [106].

A trial comparing the effectiveness of cognitive rehabilitation and psychoeducation on anxiety, depression, and ‘BPD’ symptoms, found that both interventions yielded comparable effectiveness [97]. Another study found that 10 sessions of art therapy were effective in reducing psychiatric symptom severity at EOT (*g* = 1.23, 95% CI: 0.66, 1.79) and 5-week follow-up (*g* = 1.35, 95% CI: 0.77 to 1.92) compared to waitlist [83].

### Meta-regression analysis

The results indicated that there was no significant association between the types of intervention (CBT, Psychodynamic Treatments, DBT, MBT & Schema Therapy) and intervention effectiveness for any of the outcomes examined (Table 3). Meta-regressions for the moderating effect of service setting found no significant association. However, the results showed that there is significant association between diagnoses and the effectiveness of the interventions for depressive symptoms. Studies including participants with any ‘personality disorder’ reported worse outcomes after treatment compared to studies including a ‘BPD’ only sample on depressive symptoms (*b* = − 0.71, 95% CI: − 1.39 to − 0.03, *p* = 0.04). Diagnosis was not associated with the effectiveness of the interventions for the other outcomes examined.

**Table 3:** Meta-regression analysis of intervention type, service setting, treatment duration, treatment intensity and diagnoses

Outcome	K	Variable	Beta*	95% CI	*p*-value
Anxiety symptoms	16	Intervention type ^a^			
		Psychodynamic Therapy	−0.47	−1.43, 0.49	0.33
		DBT	0.31	−0.60, 1.22	0.51
		MBT	0.18	−0.78, 1.14	0.72
		Service setting ^b^			
		Specialist day service	0.98	−0.23, 2.20	0.11
		Specialist team	0.40	−1.02, 1.83	0.58
		Standalone outpatient intervention	0.25	−0.67, 1.16	0.60
		Treatment duration ^c^			
		4–6 months	−0.63	−1.36, 0.10	0.09
		7–12 months	−0.80	−1.61, 0.01	0.05
		> 12 months	−0.26	−1.25, 0.73	0.60
		Treatment intensity ^d^			
		2–3 sessions	−0.11	−0.75, 0.54	0.74
		> 3 sessions	0.68	−0.37, 1.73	0.20
		Diagnoses ^e^			
		Other PDs	−0.80	−1.76, 0.17	0.10
Depressive symptoms	23	Intervention type			
		Psychodynamic Therapy	−0.02	−0.82, 0.77	0.95
		DBT	0.12	−0.41, 0.64	0.67
		MBT	0.51	−0.14, 1.15	0.12
		Service setting			
		Specialist day service	0.74	−0.16, 1.63	0.11
		Specialist team	0.16	−0.60, 0.93	0.68
		Standalone outpatient intervention	0.31	−0.41, 1.02	0.40
		Treatment duration			
		4–6 months	−0.27	−0.79, 0.25	0.31
		7–12 months	−0.55	−1.10, 0.01	0.05
		> 12 months	0.10	−0.60, 0.79	0.80
		Treatment intensity			
		2–3 sessions	−0.11	−0.58, 0.37	0.66
		> 3 sessions	0.03	−0.59, 0.64	0.93
		Diagnoses			
		Other PDs	−0.71	−1.39, −0.03	0.04*
Psychiatric symptoms	36	Intervention type			
		Psychodynamic Therapy	−0.04	−0.48, 0.39	0.85
		DBT	−0.08	−0.51, 0.35	0.72
		MBT	0.09	−0.45, 0.62	0.75
		Schema Therapy	0.57	−0.47, 1.60	0.28
		Service setting			
		Specialist day service	0.08	−0.51, 0.68	0.78
		Specialist team	0.24	−0.25, 0.74	0.34
		Standalone outpatient intervention	0.23	−0.19, 0.65	0.29
		Treatment duration			
		4–6 months	−0.10	−0.54, 0.33	0.64
		7–12 months	−0.24	−0.60, 0.12	0.20
		> 12 months	0.00	−0.45, 0.44	0.98
		Treatment intensity			
		2–3 sessions	0.04	−0.26, 0.35	0.79
		> 3 sessions	−0.17	−0.52, 0.17	0.32
		Diagnoses			
		Other diagnoses/symptoms	−0.05	−0.94, 0.83	0.91
		Other PDs	0.03	−0.27, 0.32	0.86
BPD symptoms	23	Intervention type			
		Psychodynamic Therapy	−0.16	−0.74, 0.42	0.58
		DBT	−0.14	−0.60, 0.33	0.57
		MBT	0.13	−0.54, 0.79	0.71
		Schema Therapy	0.82	−0.24, 1.89	0.13
		Service setting			
		Specialist day service	−0.21	−1.24, 0.82	0.69
		Specialist team	0.40	−0.78, 1.59	0.51
		Standalone outpatient intervention	−0.08	−0.93, 0.77	0.86
		Treatment duration			
		4–6 months	−0.59	−0.98, −0.20	0.003**
		7–12 months	−0.50	−0.90, −0.11	0.01*
		> 12 months	−0.10	−0.65, 0.44	0.71
		Treatment intensity			
		2–3 sessions	−0.05	−0.44, 0.33	0.78
		> 3 sessions	0.02	−0.44, 0.49	0.92
		Diagnoses			
		Other PDs	−0.41	−1.12, 0.30	0.26

*Adjusted for control group category. ** *p* < 0.05. *** *p* < 0.01. The reference group is ^a^ CBT, ^b^ Generic mental health service, ^c^ < 4 months, ^d^ < 2 sessions, and ^e^ BPD for intervention category, service setting, treatment duration, treatment intensity and diagnoses respectively. *Abbreviations*: *ΜΒΤ* Mentalization-Based Treatment, *CBT* Cognitive Behavioral Therapies, *BPD* Borderline Personality Disorder, *DBT* Dialectical Behavior Therapy, *PD* Personality Disorder

**Fig. 4:**
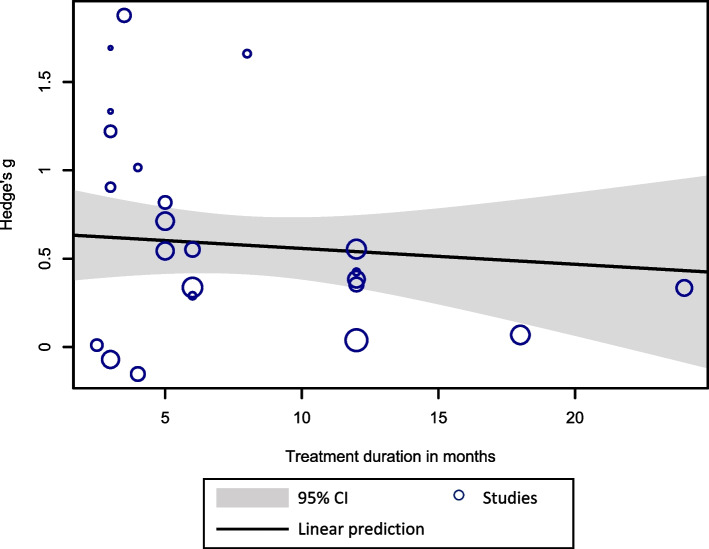
Bubble plot with a fitted meta-regression line of the influence of treatment duration on the effectiveness of community treatments for ‘BPD’ symptoms

In terms of treatment duration, the results were close to achieving statistical significance, supporting the association between treatment duration and effectiveness of interventions on anxiety and depressive symptoms. Participants who received treatments that lasted 7–12 months reported worse outcomes in anxiety symptoms (*b* = − 0.80, 95% CI: − 1.61 to 0.01, *p* = 0.05) and depressive symptoms (*b* = 0.55, 95% CI: − 1.10 to 0.01, *p* = 0.05) compared to those receiving less than 4 months of treatment. Moreover, treatments that lasted 4–6 months and 7–12 months were less effective in reducing ‘BPD’ symptom severity compared to treatments lasting less than 4 months (*b* = − 0.59, 95% CI: − 0.98 to − 0.20, *p* = 0.003 and *b* = − 0.50, 95% CI: − 0.90 to − 0.11, *p* = 0.01, respectively). We performed additional meta-regression analyses, where treatment duration was included as a continuous moderator. In this case, the moderating effect of treatment duration ceased to be significant for ‘BPD’ symptoms (*b* = − 0.01, 95% CI: − 0.04 to 0.02, *p* = 0.56), depressive symptoms (*b* = 0.01, 95% CI: − 0.03 to 0.04, *p* = 0.79) and anxiety symptoms (*b* = − 0.01, 95% CI: − 0.06 to 0.05, *p* = 0.82). Figure 4 illustrates the bubble plot of the regression line for ‘BPD’ symptoms. No association between treatment duration and the effectiveness of the interventions for psychiatric symptoms was detected. Treatment intensity was not associated with the effectiveness of the interventions for any of the assessed outcomes.

## Discussion

This systematic review and meta-analysis aimed to examine the effectiveness of a wide range of interventions provided in community settings or outpatient facilities for people with a diagnosis of ‘personality disorder’. This review is the first to systematically examine trials conducted on participants with a ‘personality disorder’ diagnosis beyond ‘BPD’ and people without a formal diagnosis but relevant symptoms and examine a variety of outcomes not previously investigated in this population. Moreover, multiple moderators that could potentially impact the efficacy of the interventions were examined.

In total, 54 RCTs were included in this review. The results indicated that community interventions were effective in treating ‘BPD’ symptoms, anxiety symptoms, depressive symptoms, and global psychiatric symptoms compared to non-active comparisons at EOT. The effectiveness of the interventions was maintained longer term (7 months after the end of treatment) for some outcomes, particularly for psychiatric symptoms and ‘BPD’ symptoms. There was weak evidence supporting the efficacy of the interventions in reducing general ‘personality disorder’ symptoms and promoting recovery at follow-up.

The results from meta-regression analysis indicated that community treatments are effective for the outcomes assessed, regardless of the type of intervention investigated (e.g. CBT, Psychodynamic treatments, DBT, MBT and Schema Therapy). More specifically, CBT, which was the most intensively studied treatment, psychodynamic treatments and DBT were found to significantly ameliorate an array of outcomes that were assessed. Although it was not possible to conduct a meta-analysis due to the limited data, individual studies examining MBT [60–62, 88], and Schema Therapy [58, 77], indicated that both treatments are effective in reducing ‘personality disorder’ symptoms. Other treatments, such as Democratic Therapeutic Community treatment [98], abandonment therapy [55], and brief adaptive therapy [106] were also found to be beneficial for people with CEN. However, further trials need to examine the efficacy of those interventions in the future. Interestingly, a trial found that Art therapy [83] was effective in treating global psychiatric symptoms in this population. Accumulative evidence suggests that art therapy can mitigate ‘personality disorder’ symptoms and increase well-being in people with CEN through different mechanisms [107].

The results from this meta-analysis are consistent with the findings from the recent Cochrane systematic review [32], regarding the efficacy of psychological interventions for ‘BPD’ symptom severity and depressive symptoms for people diagnosed with ‘BPD’. However, the present study included not only psychological interventions but also treatments provided as part of a day-treatment programme (e.g. Democratic Therapy) or other psychosocial interventions (e.g. Art Therapy) and included studies with participants diagnosed with ‘personality disorder(s)’ beyond ‘BPD’. Also, the current review examined anxiety symptoms and general psychiatric symptoms as outcomes as well, providing evidence for the efficacy of the interventions for additional outcomes. The results from this study also partially support the findings from a previous review examining psychotherapies for ‘BPD’ [27], which found that psychotherapies, particularly DBT and psychodynamic approaches were effective in treating ‘BPD’ symptoms. However, our review found larger effect sizes for the efficacy of DBT and psychodynamic therapy for ‘BPD’ symptoms, while, in contrast with the former review we found CBT to be effective for this specific outcome. This divergence in the findings might reflect the characteristics of the studies included in this review (e.g., different populations and outcome measures for ‘BPD’) and the control comparisons of the interventions which were assessed. The effectiveness of specialized psychotherapies (DBT, MBT, TFP and Schema Therapy) was also reported by a recent systematic review [31], which found that psychotherapies are effective in reducing ‘BPD’ symptoms. This study adds to the previous evidence suggesting that psychological and psychosocial interventions delivered in community and outpatient settings are not only highly effective in treating ‘BPD’ symptoms but can also improve a wide range of psychiatric symptoms in this population, with considerable maintenance across time. This is in line with previous evidence suggesting that ‘BPD’ symptoms are strongly associated with an array of psychiatric symptoms comprising the general psychopathology factor [108], thus raising the need to deliver interventions targeting global symptomatology, beyond ‘BPD’ symptoms.

The efficacy of CBT for anxiety and depressive symptoms compared to TaU/WL is in line with considerable literature supporting the effectiveness of this intervention for the aforementioned symptoms [109–111]. This is the first study to systematically examine those outcomes for people with CEN. Previous reviews have also found that CBT can effectively treat a wide spectrum of psychiatric conditions [112, 113], including ‘BPD’ symptoms [28]. The effectiveness of psychodynamic treatments for people diagnosed with ‘personality disorder’ has also been documented in the literature. Recent reviews have found that psychodynamic treatments are superior to control groups in treating ‘personality disorder’ symptoms and general psychiatric symptoms in people diagnosed with ‘personality disorder’ [26, 27]. This review expands on existing literature by supporting the efficacy of this intervention for global psychiatric symptoms manifested by patients with CEN, as well as for depressive and ‘BPD’ symptoms. The effectiveness of DBT for ‘BPD’ is well evidenced in the literature [27, 114]. This review adds to the previous studies suggesting that this intervention can be effective for other psychiatric symptoms (e.g., anxiety symptoms) in people with CEN. It is possible that improvements in the core symptomatology of ‘personality disorder’ lead to the reduction of the severity of other psychiatric symptoms (e.g., depression and anxiety). Although meta-analysis could not be conducted for studies on MBT, the effectiveness that emerged from two trials is consistent with previous reviews [34]. However, more studies are needed for firm conclusions to be drawn on the efficacy of this intervention. Finally, although the number of studies was small and meta-analysis could not be conducted, accumulative evidence suggests that Schema therapy is beneficial for people with a diagnosis of ‘BPD’ and those being diagnosed with cluster C ‘personality disorders’ or ‘narcissistic personality disorder’ [58, 77, 115]. Although the effectiveness of the major psychotherapeutic types delivered to people experiencing CEN has been widely investigated, trials examining treatments for people with CEN and major psychiatric comorbidities (e.g. Depression) are limited, while studies for specific age groups (e.g., older patients) have been rarely conducted.

The results from the meta-regression analysis provide insight into the moderating effects of different factors that can potentially impact the effectiveness of treatments for an array of outcomes, something that has not been investigated by previous studies. The equal effectiveness of the psychotherapeutic approaches examined is consistent with previous studies that could not detect significant differences between treatment types in reducing ‘BPD’ symptoms or depressive symptoms [28, 31, 32], and provides new evidence for other psychiatric symptoms as well. Interestingly, interventions delivered to participants with any ‘personality disorder’ diagnosis were less effective in treating depressive symptoms compared to ‘BPD’-only studies, while there was weak evidence for anxiety symptoms. This potentially reflects the focus of the existing treatments on ‘BPD’ diagnosis, while specific protocols for other ‘personality disorder’ diagnoses (except for ‘antisocial personality disorder’) have not been developed. Furthermore, it could be possible that ‘personality disorders’ other than ‘BPD’ are more difficult to treat with the existing interventions. In terms of treatment length, our results showed that treatments with differing duration were equally effective for the outcomes examined. This contrasts with previous evidence supporting the superiority of long-lasting interventions in treating ‘BPD’ symptoms [25], and sheds light on the ongoing debate concerning the importance of treatment length in treating ‘personality disorders’. Given the high symptom severity that people with ‘BPD’ may initially present with, it is possible that the first few treatment sessions lead to significant symptom reduction and stabilisation, effects that subsequently plateau in the course of the treatment. Consequently, short-term interventions can potentially be advantageous compared to long-term interventions, as they might have the same therapeutic effect while leading to reduction in health service costs and clinician time. However, given the contradicting nature of our findings, the latter should be treated with caution, and further well-designed studies comparing duration of treatment are required to draw firm conclusions regarding the role of treatment length in intervention effectiveness.

Although treatments delivered in the community can effectively treat ‘personality disorder’ symptoms, alongside global psychopathology, less is known about the ability of those treatments to change core personality and improve functioning. While some evidence suggests that personality traits can change following intervention [116], studies examining personality change as a treatment outcome in people with ‘personality disorder’ diagnosis are limited. In addition, little evidence is available regarding the efficacy of psychological and psychosocial treatments in improving functioning in this patient group. Therefore, we propose a shift in research towards incorporating additional treatment outcomes, such as those stated above.

### Limitations of the study

This study has several limitations. First, the majority of the studies were conducted on patients diagnosed with ‘BPD’. A consequence is that most trials included mainly female participants, with only a few RCTs including a comparable number of male participants. Also, participants of ethnic minority groups were underrepresented in the samples examined. Second, the quality of the studies was generally low to moderate, indicating high risk of bias in some cases. The latter might have had an impact on the effect estimate, leading to the overestimation of the effect sizes [117, 118]. In addition, indications of publication bias were present for most outcomes. Third, high heterogeneity was evident in some of the meta-analyses, although steps were taken to try and reduce this through separate examination of interventions and control comparisons. Our exploration of heterogeneity also did not show significant influence of a number of treatment factors, suggesting that participant characteristics may instead play a key role. Most studies included small samples, which can potentially lead to sample error [119]. It should be noted that non-English studies were excluded from this study, which might have introduced bias [120]. Furthermore, although our analysis was pairwise, comparing each intervention to a control condition, there is the possibility that variations in baseline symptom severity moderated the outcomes. Another potential limitation is the exclusion of non-randomized studies. Although this was decided to ensure that high-quality studies are included in the meta-analysis, other study designs can provide significant and clinically meaningful evidence. Our recent scoping review, which examined the treatments available for CEN, included studies with multiple study designs [44]. Lastly, a number of studies were excluded for testing modified or partial forms of already established psychotherapies (e.g., DBT). Although this significantly reduced the heterogeneity within the intervention categories, the efficacy of the former psychotherapeutic interventions was not assessed in this meta-analysis. In line with our recent scoping review [44], only a limited range of interventions were identified in the literature for this population, especially of trauma-informed treatments.

### Clinical implications

This study has significant implications for clinical practice. The results indicate that interventions are superior to TAU or no treatment in treating ‘personality disorder’ symptoms and other symptoms associated with this diagnosis. Therefore, people with CEN should be provided with ready access to specialized community treatment. Given the comparable efficacy of CBT, DBT, and psychodynamic treatments, all could be provided in community settings, while further research investigating which treatments work for which people in which settings would be valuable. The current evidence suggests that Schema Therapy could be offered for people diagnosed with cluster B and cluster C ‘personality disorder’, and MBT could be provided to people diagnosed with ‘BPD’. However, further evidence is needed to establish the effectiveness of the latter interventions. Structured and time-limited treatments, such as CBT, besides being effective for several psychiatric conditions, are also efficient. Given the effectiveness of this therapeutic approach in treating ‘personality disorder’ symptoms, CBT can be offered as a first-step intervention in primary care settings. Complex cases, or those not responding to CBT, could be referred to secondary care services, which can provide high-intensity and specialized interventions. Further development and testing of stepped-care intervention models would also be valuable for this population. This would involve informing primary care practitioners about the difficulties people who receive a ‘personality disorder’ diagnosis may experience and providing training in the assessment and initial treatment of this condition. Lastly, services should reorganize their facilities to meet the patients’ needs by providing them with more autonomy and decision making, informing them about each intervention’s qualities and characteristics (i.e. duration, intensity, treatment style, working model), and enabling them to choose between treatments that show similar effectiveness [98].

### Future directions

In terms of future research, further high-quality trials with larger samples need to be conducted, which will measure outcomes at different follow-up time points to investigate long-term effectiveness. Some suggestions for improving the overall quality of the trials are the blinding of the assessors, the use of genuine randomization methοds (e.g., permuted block randomization), the use of appropriate and clear subject selection criteria (e.g., established diagnosis of ‘personality disorder’ and examination of potential comorbidities), and the adherence to a published intervention protocol. Importantly, valid scales measuring core ‘personality disorder’ symptoms and other psychiatric symptoms, should be used universally for the measurement of each outcome to facilitate the comparison of intervention effects between trials. Another significant methodological necessity would be to develop and consistently use standardized control comparison interventions (e.g. manualized supportive therapy) in trials examining psychological interventions, in order for the comparisons to be comparable across different studies. Researchers who conduct each study should not be involved in the delivery of any treatment and instead use independent clinicians or therapists. The examination of existing interventions for different types of ‘personality disorders’ is necessary to shed light on potentially effective therapeutic interventions for ‘personality disorders’ beyond ‘BPD’. Moreover, future trials should investigate: a) the effectiveness of understudied psychosocial interventions, such as peer support and of different approaches to designing services, b) the effectiveness of different interventions across populations, including younger and older people and people of minority ethnic groups, and c) outcomes valued by service users, such as loneliness, occupational status, quality of life, and treatment targets that matter to them (e.g., trauma). Further developments of existing interventions involving the contribution of service users is also essential for the improvement of the treatment that the latter group receives. Finally, more research is needed to explore the mechanisms of change [19, 121].

## Conclusions

In conclusion, this systematic review and meta-analysis provides evidence that psychological and psychosocial treatments offered in community or outpatient settings are beneficial for people experiencing ‘personality disorder’ symptoms and can significantly reduce a range of psychiatric symptoms, including symptoms of ‘BPD’. Community mental health practitioners can utilize the psychological interventions available to treat people showing symptoms associated with a ‘personality disorder’ diagnosis. Mental health guidelines need to highlight the effectiveness of treatments provided in the community for this population, while stepped-care interventions should be developed and delivered in community settings. Challenging therapeutic pessimism associated with this patient group is equally important, considering the effectiveness of the interventions and patients’ engagement with those treatments.

## Supplementary Information

**Additional file 1.**

**Additional file 2.**

**Additional file 3.**

**Additional file 4.**

## Data Availability

All data generated or analysed during this study are included in this published article (Additional file 4).

## Abbreviations

EMBASE: Excerpta Medica dataBASE
CINAHL: Cumulative Index to Nursing and Allied Health Literature
HMIC: Health Management Information Consortium
ASSIA: Applied Social Sciences Index and Abstracts
RCT: Randomized Control Trial
BPD: Borderline Personality Disorder
CEN: Complex Emotional Needs
CBT: Cognitive Behavioural Therapy
DBT: Dialectical Behaviour Therapy
MBT: Mentalization-Based Therapy
TFT: Transference-Focused Therapy
PTSD: Post-Traumatic Stress Disorder
PRISMA: Preferred Reporting Items for Systematic Reviews and Meta-Analyses
PROSPERO: International Prospective Register of Systematic Reviews
TAU: Treatment As Usual
WL: Waitlist
SMD: Standardised Mean Difference
EOT: End Of Treatment
CI: Confidence Interval
ACT: Acceptance and Commitment Therapy
CAT: Cognitive Analytic Therapy
STEPPS: Systems Training for Emotional Predictability and Problem Solving
BSI: Brief Symptom Inventory
CGI: Clinical Global Impression
HRSD: Hamilton Rating Scale for Depression
BDI: Beck’s Depression Inventory
SCL-90: Symptom Checklist 90
SCID-II: Structured Clinical Interview for DSM-IV Axis II Disorders
STAI: State-Trait Anxiety Inventory
ZAN-BPD: Zanarini Rating Scale for Borderline Personality Disorder
MCMI: Millon Clinical Multiaxial Inventory
HAM-A: Hamilton Anxiety Rating Scale
MADRS: Montgomery-Åsberg Depression Rating Scale
BPRS: Brief Psychiatric Rating Scale
CORE-OM: Clinical Outcomes in Routine Evaluation-Outcome Measure
GHQ: General Health Questionnaire
BSL-23: Borderline Symptom List
BEST: Borderline Evaluation of Severity over Time
DASS-(A/D): Depression Anxiety Stress Scales (Anxiety/ Depression)
BPDSI: Borderline Personality Disorder Severity index
OQ-45: Outcome Questionnaire 45
LWASQ: Lehrer Woolfolk Anxiety Symptom Questionnaire
HADS-(A/D): Hospital Anxiety and Depression Scale (Anxiety/ Depression)
PD: Personality Disorder
