# Supplementary material for: Effectiveness of outpatient and community treatments for people with a diagnosis of ‘personality disorder’: systematic review and meta-analysis
Source: BMC Psychiatry. 2023 Jan 21;23:57. doi: 10.1186/s12888-022-04483-0 (PMC9862782; doi:10.1186/s12888-022-04483-0)
Supplement: Supplementary file 1 — Additional file 1. [file 12888_2022_4483_MOESM1_ESM.docx]

**Table S1.**

Database: Ovid MEDLINE(R) and Epub Ahead of Print, In-Process & Other Non-Indexed Citations and Daily

Host: Ovid

Data Parameters: 1946 to December 10, 2019

Date of search: Wednesday 11^th^ December 2019

Search strategy:

Search Strategy:

| **#** | **Searches** | **Results** |
| --- | --- | --- |
| 1 | exp *Personality Disorders/ | 28263 |
| 2 | ((personality or character*) adj3 disorder$).ti,ab,kw. | 65164 |
| 3 | "axis II".ti,ab,kw. | 1959 |
| 4 | ("Complex trauma" or CPTSD or "complex post-traumatic stress disorder").ti,ab,kw. | 535 |
| 5 | (Complex adj (needs or mental)).ti,ab,kw. | 1929 |
| 6 | *Self-Injurious Behavior/ | 5465 |
| 7 | (Self-harm or self-injury).ti,ab,kw. | 7484 |
| 8 | (emotion* adj2 (regulation or dysregulation or unstable or instability)).ti,ab,kw. | 10588 |
| 9 | mood instability.ti,ab,kw. | 254 |
| 10 | 1 or 2 or 3 or 4 or 5 or 6 or 7 or 8 or 9 | 103674 |
| 11 | Community Health Services/ | 31050 |
| 12 | Community Mental Health Services/ | 18317 |
| 13 | ((commun$ adj5 (mental health or model$1 or pathway$1 or program$ or evaluat$ or intervention$ or implement$)) or camhs or cmht$1).ti,ab,kw. | 79584 |
| 14 | (community adj5 (agenc$ or care or center$ or centre$ or clinic$ or consultant$ or doctor$ or employee$ or expert$ or facilitator$ or healthcare or instructor$ or leader$ or manager$ or mentor$ or nurs$ or personnel$ or pharmacy or pharmacist$ or psychiatrist$ or psychologist$ or psychotherapist$ or specialist$ or skill$ or staff$ or team$ or therapist$ or tutor$ or visit$ or worker$ or group$ or independent or (peer$ adj3 support$) or survivor or outpatient$ or "out patient$")).ti,ab,kw. | 96749 |
| 15 | (commun$ adj5 (service or hub$ or based or deliver$ or interact$ or led or maintenance or mediat$ or operated or provides or provider$ or run or setting$ or support or rehab$ or therap$ or service$ or treatment or management or assessment or assistance or care or day or week)).ti,ab,kw. | 205151 |
| 16 | (Independent sector or ((non institutional$ or noninstitution$) adj2 (sector$ or setting$))).ti,ab,kw. | 367 |
| 17 | (network or outreach or ((specialist or day or whole) adj3 service)).ti,ab,kw. | 351098 |
| 18 | ((treatment* or (Dialectical behavior therapy or Dialectical behaviour therapy or DBT) or Psychotherapy* or specialist or psychiatry* or therapeutic or day or outreach or therap*) adj3 (Outpatient* or community or Service* or Center* or Centre* or Clinic*1 or Team* or program* or provider* or practice or setting* or care or community or unit* or hospital*)).ti,ab,kw. | 244595 |
| 19 | 11 or 12 or 13 or 14 or 15 or 16 or 17 or 18 | 851066 |
| 20 | Interview*.af. | 373632 |
| 21 | Experience*.af. | 1028333 |
| 22 | qualitative.tw. | 212806 |
| 23 | Qualitative Research/ | 50164 |
| 24 | 20 or 21 or 22 or 23 | 1444445 |
| 25 | randomized controlled trial.pt. | 495635 |
| 26 | controlled clinical trial.pt. | 93449 |
| 27 | (randomized or randomised).ab. | 553632 |
| 28 | placebo.ab. | 203251 |
| 29 | clinical trials as topic.sh. | 189357 |
| 30 | randomly.ab. | 322897 |
| 31 | trial.ti. | 209094 |
| 32 | 25 or 26 or 27 or 28 or 29 or 30 or 31 | 1289520 |
| 33 | Epidemiologic studies/ | 8156 |
| 34 | exp case control studies/ | 1037554 |
| 35 | Case control.tw. | 120110 |
| 36 | (cohort adj (study or studies)).tw. | 190004 |
| 37 | Cohort analy$.tw. | 7484 |
| 38 | (Follow up adj (study or studies)).tw. | 47969 |
| 39 | (observational adj (study or studies)).tw. | 98982 |
| 40 | Longitudinal.tw. | 232846 |
| 41 | Retrospective.tw. | 497909 |
| 42 | Cross sectional.tw. | 329612 |
| 43 | Cross-sectional studies/ | 311409 |
| 44 | 33 or 34 or 35 or 36 or 37 or 38 or 39 or 40 or 41 or 42 or 43 | 2028189 |
| 45 | 32 or 44 | 3197720 |
| 46 | "Surveys and Questionnaires"/ | 443562 |
| 47 | survey$.tw. | 612248 |
| 48 | exp clinical pathway/ | 6469 |
| 49 | exp clinical protocol/ | 163100 |
| 50 | exp consensus/ | 11712 |
| 51 | exp consensus development conference/ | 11685 |
| 52 | exp consensus development conferences as topic/ | 2772 |
| 53 | critical pathways/ | 6469 |
| 54 | exp guideline/ | 33000 |
| 55 | guidelines as topic/ | 38818 |
| 56 | exp practice guideline/ | 26125 |
| 57 | health planning guidelines/ | 4067 |
| 58 | (guideline or practice guideline or consensus development conference or consensus development conference, NIH).pt. | 42151 |
| 59 | (position statement* or policy statement* or practice parameter* or best practice*).ti,ab,kf,kw. | 31048 |
| 60 | (standards or guideline or guidelines).ti,kf,kw. | 105440 |
| 61 | ((practice or treatment* or clinical) adj guideline*).ab. | 37832 |
| 62 | (CPG or CPGs).ti. | 5569 |
| 63 | consensus*.ti,kf,kw. | 24689 |
| 64 | consensus*.ab. /freq=2 | 23911 |
| 65 | ((critical or clinical or practice) adj2 (path or paths or pathway or pathways or protocol*)).ti,ab,kf,kw. | 19229 |
| 66 | recommendat*.ti,kf,kw. | 39030 |
| 67 | (care adj2 (standard or path or paths or pathway or pathways or map or maps or plan or plans)).ti,ab,kf,kw. | 55156 |
| 68 | (algorithm* adj2 (screening or examination or test or tested or testing or assessment* or diagnosis or diagnoses or diagnosed or diagnosing)).ti,ab,kf,kw. | 7192 |
| 69 | (algorithm* adj2 (pharmacotherap* or chemotherap* or chemotreatment* or therap* or treatment* or intervention*)).ti,ab,kf,kw. | 9314 |
| 70 | 46 or 47 or 48 or 49 or 50 or 51 or 52 or 53 or 54 or 55 or 56 or 57 or 58 or 59 or 60 or 61 or 62 or 63 or 64 or 65 or 66 or 67 or 68 or 69 | 1422311 |
| 71 | (systematic adj3 review$).ti,ab,kw. | 164344 |
| 72 | 24 or 45 or 70 or 71 | 5190597 |
| 73 | 10 and 19 and 72 | 3984 |

**Table S2.**

Database: Embase

Host: Ovid

Data Parameters: 1980 to 2019 Week 49

Date of search: Wednesday 11^th^ December 2019

Search strategy:

| **#** | **Searches** | **Results** |
| --- | --- | --- |
| 1 | exp *personality disorder/ | 26184 |
| 2 | ((personality or character*) adj3 disorder$).ti,ab,kw. | 92931 |
| 3 | "axis II".ti,ab,kw. | 2594 |
| 4 | ("Complex trauma" or CPTSD or "complex post-traumatic stress disorder").ti,ab,kw. | 661 |
| 5 | (Complex adj (needs or mental)).ti,ab,kw. | 2711 |
| 6 | *automutilation/ | 7119 |
| 7 | (Self-harm or self-injury).ti,ab,kw. | 9852 |
| 8 | (emotion* adj2 (regulation or dysregulation or unstable or instability)).ti,ab,kw. | 14860 |
| 9 | "mood instability".ti,ab,kw. | 471 |
| 10 | 1 or 2 or 3 or 4 or 5 or 6 or 7 or 8 or 9 | 134437 |
| 11 | *community care/ | 18783 |
| 12 | *mental health service/ | 24783 |
| 13 | ((commun$ adj5 (mental health or model$1 or pathway$1 or program$ or evaluat$ or intervention$ or implement$)) or camhs or cmht$1).ti,ab,kw. | 97864 |
| 14 | (commun$ adj5 (service or hub$ or based or deliver$ or interact$ or led or maintenance or mediat$ or operated or provides or provider$ or run or setting$ or support or rehab$ or therap$ or service$ or treatment or management or assessment or assistance or care or day or week)).ti,ab,kw. | 263482 |
| 15 | (community adj5 (agenc$ or care or center$ or centre$ or clinic$ or consultant$ or doctor$ or employee$ or expert$ or facilitator$ or healthcare or instructor$ or leader$ or manager$ or mentor$ or nurs$ or personnel$ or pharmacy or pharmacist$ or psychiatrist$ or psychologist$ or psychotherapist$ or specialist$ or skill$ or staff$ or team$ or therapist$ or tutor$ or visit$ or worker$ or group$ or independent or (peer$ adj3 support$) or survivor or outpatient$ or "out patient$")).ti,ab,kw. | 129041 |
| 16 | (Independent sector or ((non institutional$ or noninstitution$) adj2 (sector$ or setting$))).ti,ab,kw. | 406 |
| 17 | (network or outreach or ((specialist or day or whole) adj3 service)).ti,ab,kw. | 442567 |
| 18 | ((treatment* or (Dialectical behavior therapy or Dialectical behaviour therapy or DBT) or Psychotherapy* or specialist or psychiatry* or therapeutic or day or outreach or therap*) adj3 (Outpatient* or community or Service* or Center* or Centre* or Clinic*1 or Team* or program* or provider* or practice or setting* or care or community or unit* or hospital*)).ti,ab,kw. | 365376 |
| 19 | 11 or 12 or 13 or 14 or 15 or 16 or 17 or 18 | 1115235 |
| 20 | Interview*.af. | 471643 |
| 21 | Experience*.af. | 1409073 |
| 22 | qualitative.tw. | 258870 |
| 23 | Qualitative Research/ | 69740 |
| 24 | 20 or 21 or 22 or 23 | 1921768 |
| 25 | random$.af. | 1679449 |
| 26 | Clinical study/ | 109718 |
| 27 | Case control study/ | 149438 |
| 28 | Family study/ | 25901 |
| 29 | Longitudinal study/ | 133160 |
| 30 | Retrospective study/ | 852942 |
| 31 | Prospective study/ | 566063 |
| 32 | Randomized controlled trials/ | 170502 |
| 33 | 31 not 32 | 560185 |
| 34 | Cohort analysis/ | 529867 |
| 35 | (Cohort adj (study or studies)).mp. | 284531 |
| 36 | (Case control adj (study or studies)).tw. | 128035 |
| 37 | (follow up adj (study or studies)).tw. | 58655 |
| 38 | (observational adj (study or studies)).tw. | 156172 |
| 39 | (epidemiologic$ adj (study or studies)).tw. | 100782 |
| 40 | (cross sectional adj (study or studies)).tw. | 202498 |
| 41 | 25 or 26 or 27 or 28 or 29 or 30 or 33 or 34 or 35 or 36 or 37 or 38 or 39 or 40 | 3924408 |
| 42 | "Surveys and Questionnaires"/ | 656595 |
| 43 | survey$.tw. | 759236 |
| 44 | exp clinical pathway/ | 8254 |
| 45 | exp clinical protocol/ | 96225 |
| 46 | exp consensus/ | 66046 |
| 47 | exp consensus development conference/ | 23930 |
| 48 | exp consensus development conferences as topic/ | 23930 |
| 49 | critical pathways/ | 8254 |
| 50 | exp practice guideline/ | 523652 |
| 51 | health planning guidelines/ | 86081 |
| 52 | (guideline or practice guideline or consensus development conference or consensus development conference, NIH).pt. | 0 |
| 53 | (position statement* or policy statement* or practice parameter* or best practice*).ti,ab,kw. | 45154 |
| 54 | (standards or guideline or guidelines).ti,kw. | 143243 |
| 55 | ((practice or treatment* or clinical) adj guideline*).ab. | 57353 |
| 56 | (CPG or CPGs).ti. | 6707 |
| 57 | consensus*.ti,kw. | 30932 |
| 58 | consensus*.ab. /freq=2 | 31634 |
| 59 | ((critical or clinical or practice) adj2 (path or paths or pathway or pathways or protocol*)).ti,ab,kw. | 29960 |
| 60 | recommendat*.ti,kw. | 48332 |
| 61 | (care adj2 (standard or path or paths or pathway or pathways or map or maps or plan or plans)).ti,ab,kw. | 96662 |
| 62 | (algorithm* adj2 (screening or examination or test or tested or testing or assessment* or diagnosis or diagnoses or diagnosed or diagnosing)).ti,ab,kw. | 9924 |
| 63 | (algorithm* adj2 (pharmacotherap* or chemotherap* or chemotreatment* or therap* or treatment* or intervention*)).ti,ab,kw. | 14123 |
| 64 | 42 or 43 or 44 or 45 or 46 or 47 or 48 or 49 or 50 or 51 or 52 or 53 or 54 or 55 or 56 or 57 or 58 or 59 or 60 or 61 or 62 or 63 | 2131726 |
| 65 | (systematic adj3 review$).ti,ab,kw. | 208956 |
| 66 | 24 or 41 or 64 or 65 | 6771882 |
| 67 | 10 and 19 and 66 | 6002 |

**Table S3.**

Database: Social Policy and Practice

Host: Ovid

Data Parameters: 201910

Date of search: Wednesday 11^th^ December 2019

Search strategy:

| **#** | **Searches** | **Results** |
| --- | --- | --- |
| 1 | ((personality or character*) adj3 disorder$).ti,ab. | 998 |
| 2 | "axis II".ti,ab. | 22 |
| 3 | ("Complex trauma" or CPTSD or "complex post-traumatic stress disorder").ti,ab. | 74 |
| 4 | (Complex adj (needs or mental)).ti,ab. | 1178 |
| 5 | (Self-harm or self-injury).ti,ab. | 1183 |
| 6 | (emotion* adj2 (regulation or dysregulation or unstable or instability)).ti,ab. | 310 |
| 7 | "mood instability".ti,ab. | 1 |
| 8 | 1 or 2 or 3 or 4 or 5 or 6 or 7 | 3634 |
| 9 | ((commun$ adj5 (mental health or model$1 or pathway$1 or program$ or evaluat$ or intervention$ or implement$)) or camhs or cmht$1).ti,ab. | 8278 |
| 10 | (community adj5 (agenc$ or care or center$ or centre$ or clinic$ or consultant$ or doctor$ or employee$ or expert$ or facilitator$ or healthcare or instructor$ or leader$ or manager$ or mentor$ or nurs$ or personnel$ or pharmacy or pharmacist$ or psychiatrist$ or psychologist$ or psychotherapist$ or specialist$ or skill$ or staff$ or team$ or therapist$ or tutor$ or visit$ or worker$ or group$ or independent or (peer$ adj3 support$) or survivor or outpatient$ or "out patient$")).ti,ab. | 18140 |
| 11 | (commun$ adj5 (service or hub$ or based or deliver$ or interact$ or led or maintenance or mediat$ or operated or provides or provider$ or run or setting$ or support or rehab$ or therap$ or service$ or treatment or management or assessment or assistance or care or day or week)).ti,ab. | 27515 |
| 12 | (Independent sector or ((non institutional$ or noninstitution$) adj2 (sector$ or setting$))).ti,ab. | 556 |
| 13 | (network or outreach or ((specialist or day or whole) adj3 service)).ti,ab. | 6514 |
| 14 | ((treatment* or (Dialectical behavior therapy or Dialectical behaviour therapy or DBT) or Psychotherapy* or specialist or psychiatry* or therapeutic or day or outreach or therap*) adj3 (Outpatient* or community or Service* or Center* or Centre* or Clinic*1 or Team* or program* or provider* or practice or setting* or care or community or unit* or hospital*)).ti,ab. | 13403 |
| 15 | 9 or 10 or 11 or 12 or 13 or 14 | 49011 |
| 16 | Interview*.af. | 25584 |
| 17 | Experience*.af. | 47707 |
| 18 | qualitative.tw. | 11695 |
| 19 | 16 or 17 or 18 | 67923 |
| 20 | randomized controlled trial.pt. | 0 |
| 21 | controlled clinical trial.pt. | 0 |
| 22 | (randomized or randomised).ab. | 2322 |
| 23 | placebo.ab. | 109 |
| 24 | clinical trials as topic.sh. | 0 |
| 25 | randomly.ab. | 1240 |
| 26 | trial.ti. | 919 |
| 27 | 20 or 21 or 22 or 23 or 24 or 25 or 26 | 3841 |
| 28 | Case control.tw. | 222 |
| 29 | (cohort adj (study or studies)).tw. | 881 |
| 30 | Cohort analy$.tw. | 19 |
| 31 | (Follow up adj (study or studies)).tw. | 402 |
| 32 | (observational adj (study or studies)).tw. | 259 |
| 33 | Longitudinal.tw. | 4985 |
| 34 | Retrospective.tw. | 1017 |
| 35 | Cross sectional.tw. | 2357 |
| 36 | 28 or 29 or 30 or 31 or 32 or 33 or 34 or 35 | 9332 |
| 37 | 27 or 36 | 12906 |
| 38 | survey$.tw. | 26687 |
| 39 | (guideline or practice guideline or consensus development conference or consensus development conference, NIH).pt. | 0 |
| 40 | (position statement* or policy statement* or practice parameter* or best practice*).ti,ab. | 3454 |
| 41 | (standards or guideline or guidelines).ti. | 2820 |
| 42 | ((practice or treatment* or clinical) adj guideline*).ab. | 582 |
| 43 | (CPG or CPGs).ti. | 0 |
| 44 | consensus*.ti. | 135 |
| 45 | consensus*.ab. /freq=2 | 126 |
| 46 | ((critical or clinical or practice) adj2 (path or paths or pathway or pathways or protocol*)).ti,ab. | 89 |
| 47 | recommendat*.ti. | 733 |
| 48 | (care adj2 (standard or path or paths or pathway or pathways or map or maps or plan or plans)).ti,ab. | 2304 |
| 49 | (algorithm* adj2 (screening or examination or test or tested or testing or assessment* or diagnosis or diagnoses or diagnosed or diagnosing)).ti,ab. | 9 |
| 50 | (algorithm* adj2 (pharmacotherap* or chemotherap* or chemotreatment* or therap* or treatment* or intervention*)).ti,ab. | 4 |
| 51 | 38 or 39 or 40 or 41 or 42 or 43 or 44 or 45 or 46 or 47 or 48 or 49 or 50 | 35860 |
| 52 | (systematic adj3 review$).ti,ab. | 3133 |
| 53 | 19 or 37 or 51 or 52 | 103557 |
| 54 | 8 and 15 and 53 | 402 |

**Table S4.**

Database: PsycINFO

Host: Ovid

Data Parameters: 1806 to December Week 2 2019

Date of search: Wednesday 11^th^ December 2019

Search strategy:

| **#** | **Searches** | **Results** |
| --- | --- | --- |
| 1 | exp *Personality Disorders/ | 23241 |
| 2 | ((personality or character*) adj3 disorder$).ti,ab. | 43602 |
| 3 | "axis II".ti,ab. | 2674 |
| 4 | ("Complex trauma" or CPTSD or "complex post-traumatic stress disorder").ti,ab. | 860 |
| 5 | (Complex adj (needs or mental)).ti,ab. | 1897 |
| 6 | *Self-Injurious Behavior/ | 3279 |
| 7 | (Self-harm or self-injury).ti,ab. | 8008 |
| 8 | (emotion* adj2 (regulation or dysregulation or unstable or instability)).ti,ab. | 16812 |
| 9 | "mood instability".ti,ab. | 260 |
| 10 | 1 or 2 or 3 or 4 or 5 or 6 or 7 or 8 or 9 | 76125 |
| 11 | Community Health Services/ | 0 |
| 12 | Community Mental Health Services/ | 7531 |
| 13 | ((commun$ adj5 (mental health or model$1 or pathway$1 or program$ or evaluat$ or intervention$ or implement$)) or camhs or cmht$1).ti,ab. | 58711 |
| 14 | (community adj5 (agenc$ or care or center$ or centre$ or clinic$ or consultant$ or doctor$ or employee$ or expert$ or facilitator$ or healthcare or instructor$ or leader$ or manager$ or mentor$ or nurs$ or personnel$ or pharmacy or pharmacist$ or psychiatrist$ or psychologist$ or psychotherapist$ or specialist$ or skill$ or staff$ or team$ or therapist$ or tutor$ or visit$ or worker$ or group$ or independent or (peer$ adj3 support$) or survivor or outpatient$ or "out patient$")).ti,ab. | 54858 |
| 15 | (commun$ adj5 (service or hub$ or based or deliver$ or interact$ or led or maintenance or mediat$ or operated or provides or provider$ or run or setting$ or support or rehab$ or therap$ or service$ or treatment or management or assessment or assistance or care or day or week)).ti,ab. | 121630 |
| 16 | (Independent sector or ((non institutional$ or noninstitution$) adj2 (sector$ or setting$))).ti,ab. | 212 |
| 17 | (network or outreach or ((specialist or day or whole) adj3 service)).ti,ab. | 93136 |
| 18 | ((treatment* or (Dialectical behavior therapy or Dialectical behaviour therapy or DBT) or Psychotherapy* or specialist or psychiatry* or therapeutic or day or outreach or therap*) adj3 (Outpatient* or community or Service* or Center* or Centre* or Clinic*1 or Team* or program* or provider* or practice or setting* or care or community or unit* or hospital*)).ti,ab. | 126549 |
| 19 | 11 or 12 or 13 or 14 or 15 or 16 or 17 or 18 | 357869 |
| 20 | Interview*.af. | 630489 |
| 21 | Experience*.af. | 1138590 |
| 22 | qualitative.tw. | 159406 |
| 23 | Qualitative Research/ | 8543 |
| 24 | 20 or 21 or 22 or 23 | 1524734 |
| 25 | randomized controlled trial.pt. | 0 |
| 26 | controlled clinical trial.pt. | 0 |
| 27 | (randomized or randomised).ab. | 74418 |
| 28 | placebo.ab. | 38635 |
| 29 | clinical trials as topic.sh. | 0 |
| 30 | randomly.ab. | 70615 |
| 31 | trial.ti. | 29152 |
| 32 | 25 or 26 or 27 or 28 or 29 or 30 or 31 | 168398 |
| 33 | Epidemiologic studies/ | 0 |
| 34 | exp case control studies/ | 0 |
| 35 | Case control.tw. | 10553 |
| 36 | (cohort adj (study or studies)).tw. | 20487 |
| 37 | Cohort analy$.tw. | 872 |
| 38 | (Follow up adj (study or studies)).tw. | 12773 |
| 39 | (observational adj (study or studies)).tw. | 9923 |
| 40 | Longitudinal.tw. | 112758 |
| 41 | Retrospective.tw. | 34368 |
| 42 | Cross sectional.tw. | 74211 |
| 43 | Cross-sectional studies/ | 0 |
| 44 | 33 or 34 or 35 or 36 or 37 or 38 or 39 or 40 or 41 or 42 or 43 | 246843 |
| 45 | 32 or 44 | 404271 |
| 46 | "Surveys and Questionnaires"/ | 0 |
| 47 | survey$.tw. | 289751 |
| 48 | exp care pathways/ | 0 |
| 49 | exp Clinical protocols/ | 0 |
| 50 | exp Consensus conferences/ | 0 |
| 51 | critical pathways/ | 0 |
| 52 | exp Guidelines/ | 0 |
| 53 | guidelines as topic/ | 0 |
| 54 | exp Clinical guidelines/ | 0 |
| 55 | health planning guidelines/ | 0 |
| 56 | (guideline or practice guideline or consensus development conference or consensus development conference, NIH).pt. | 0 |
| 57 | (position statement* or policy statement* or practice parameter* or best practice*).ti,ab. | 16045 |
| 58 | (standards or guideline or guidelines).ti. | 12603 |
| 59 | ((practice or treatment* or clinical) adj guideline*).ab. | 7269 |
| 60 | (CPG or CPGs).ti. | 123 |
| 61 | consensus*.ti. | 3064 |
| 62 | consensus*.ab. /freq=2 | 4602 |
| 63 | ((critical or clinical or practice) adj2 (path or paths or pathway or pathways or protocol*)).ti,ab. | 1894 |
| 64 | recommendat*.ti. | 6227 |
| 65 | (care adj2 (standard or path or paths or pathway or pathways or map or maps or plan or plans)).ti,ab. | 7885 |
| 66 | (algorithm* adj2 (screening or examination or test or tested or testing or assessment* or diagnosis or diagnoses or diagnosed or diagnosing)).ti,ab. | 612 |
| 67 | (algorithm* adj2 (pharmacotherap* or chemotherap* or chemotreatment* or therap* or treatment* or intervention*)).ti,ab. | 702 |
| 68 | 46 or 47 or 48 or 49 or 50 or 51 or 52 or 53 or 54 or 55 or 56 or 57 or 58 or 59 or 60 or 61 or 62 or 63 or 64 or 65 or 66 or 67 | 339013 |
| 69 | (systematic adj3 review$).ti,ab. | 30104 |
| 70 | 24 or 45 or 68 or 69 | 1906582 |
| 71 | 10 and 19 and 70 | 5285 |

**Table S5.**

Database: HMIC

Host: Ovid

Data Parameters: 1979 to September 2019

Date of search: Wednesday 11^th^ December 2019

Search strategy:

| **#** | **Searches** | **Results** |
| --- | --- | --- |
| 1 | exp *Personality Disorders/ | 0 |
| 2 | ((personality or character*) adj3 disorder$).ti,ab. | 507 |
| 3 | "axis II".ti,ab. | 12 |
| 4 | ("Complex trauma" or CPTSD or "complex post-traumatic stress disorder").ti,ab. | 1 |
| 5 | (Complex adj (needs or mental)).ti,ab. | 364 |
| 6 | *Self-Injurious Behavior/ | 0 |
| 7 | (Self-harm or self-injury).ti,ab. | 583 |
| 8 | (emotion* adj2 (regulation or dysregulation or unstable or instability)).ti,ab. | 26 |
| 9 | "mood instability".ti,ab. | 0 |
| 10 | 1 or 2 or 3 or 4 or 5 or 6 or 7 or 8 or 9 | 1447 |
| 11 | Community Health Services/ | 1735 |
| 12 | Community Mental Health Services/ | 960 |
| 13 | ((commun$ adj5 (mental health or model$1 or pathway$1 or program$ or evaluat$ or intervention$ or implement$)) or camhs or cmht$1).ti,ab. | 5913 |
| 14 | (community adj5 (agenc$ or care or center$ or centre$ or clinic$ or consultant$ or doctor$ or employee$ or expert$ or facilitator$ or healthcare or instructor$ or leader$ or manager$ or mentor$ or nurs$ or personnel$ or pharmacy or pharmacist$ or psychiatrist$ or psychologist$ or psychotherapist$ or specialist$ or skill$ or staff$ or team$ or therapist$ or tutor$ or visit$ or worker$ or group$ or independent or (peer$ adj3 support$) or survivor or outpatient$ or "out patient$")).ti,ab. | 17606 |
| 15 | (commun$ adj5 (service or hub$ or based or deliver$ or interact$ or led or maintenance or mediat$ or operated or provides or provider$ or run or setting$ or support or rehab$ or therap$ or service$ or treatment or management or assessment or assistance or care or day or week)).ti,ab. | 22241 |
| 16 | (Independent sector or ((non institutional$ or noninstitution$) adj2 (sector$ or setting$))).ti,ab. | 713 |
| 17 | (network or outreach or ((specialist or day or whole) adj3 service)).ti,ab. | 4987 |
| 18 | ((treatment* or (Dialectical behavior therapy or Dialectical behaviour therapy or DBT) or Psychotherapy* or specialist or psychiatry* or therapeutic or day or outreach or therap*) adj3 (Outpatient* or community or Service* or Center* or Centre* or Clinic*1 or Team* or program* or provider* or practice or setting* or care or community or unit* or hospital*)).ti,ab. | 13449 |
| 19 | 11 or 12 or 13 or 14 or 15 or 16 or 17 or 18 | 43386 |
| 20 | Interview*.af. | 19889 |
| 21 | Experience*.af. | 29149 |
| 22 | qualitative.tw. | 8940 |
| 23 | Qualitative Research/ | 1278 |
| 24 | 20 or 21 or 22 or 23 | 46583 |
| 25 | randomized controlled trial.pt. | 0 |
| 26 | controlled clinical trial.pt. | 0 |
| 27 | (randomized or randomised).ab. | 6117 |
| 28 | placebo.ab. | 734 |
| 29 | clinical trials as topic.sh. | 0 |
| 30 | randomly.ab. | 2840 |
| 31 | trial.ti. | 3025 |
| 32 | 25 or 26 or 27 or 28 or 29 or 30 or 31 | 9053 |
| 33 | Epidemiologic studies/ | 122 |
| 34 | exp case control studies/ | 203 |
| 35 | Case control.tw. | 1408 |
| 36 | (cohort adj (study or studies)).tw. | 3400 |
| 37 | Cohort analy$.tw. | 144 |
| 38 | (Follow up adj (study or studies)).tw. | 640 |
| 39 | (observational adj (study or studies)).tw. | 1450 |
| 40 | Longitudinal.tw. | 3287 |
| 41 | Retrospective.tw. | 3178 |
| 42 | Cross sectional.tw. | 5012 |
| 43 | Cross-sectional studies/ | 0 |
| 44 | 33 or 34 or 35 or 36 or 37 or 38 or 39 or 40 or 41 or 42 or 43 | 16093 |
| 45 | 32 or 44 | 24059 |
| 46 | "Surveys and Questionnaires"/ | 0 |
| 47 | survey$.tw. | 28543 |
| 48 | exp care pathways/ | 1228 |
| 49 | exp Clinical protocols/ | 62 |
| 50 | exp Consensus conferences/ | 29 |
| 51 | critical pathways/ | 0 |
| 52 | exp Guidelines/ | 6914 |
| 53 | guidelines as topic/ | 0 |
| 54 | exp Clinical guidelines/ | 1494 |
| 55 | health planning guidelines/ | 0 |
| 56 | (guideline or practice guideline or consensus development conference or consensus development conference, NIH).pt. | 0 |
| 57 | (position statement* or policy statement* or practice parameter* or best practice*).ti,ab. | 2168 |
| 58 | (standards or guideline or guidelines).ti. | 5716 |
| 59 | ((practice or treatment* or clinical) adj guideline*).ab. | 1381 |
| 60 | (CPG or CPGs).ti. | 1 |
| 61 | consensus*.ti. | 353 |
| 62 | consensus*.ab. /freq=2 | 328 |
| 63 | ((critical or clinical or practice) adj2 (path or paths or pathway or pathways or protocol*)).ti,ab. | 401 |
| 64 | recommendat*.ti. | 1261 |
| 65 | (care adj2 (standard or path or paths or pathway or pathways or map or maps or plan or plans)).ti,ab. | 2812 |
| 66 | (algorithm* adj2 (screening or examination or test or tested or testing or assessment* or diagnosis or diagnoses or diagnosed or diagnosing)).ti,ab. | 26 |
| 67 | (algorithm* adj2 (pharmacotherap* or chemotherap* or chemotreatment* or therap* or treatment* or intervention*)).ti,ab. | 20 |
| 68 | 46 or 47 or 48 or 49 or 50 or 51 or 52 or 53 or 54 or 55 or 56 or 57 or 58 or 59 or 60 or 61 or 62 or 63 or 64 or 65 or 66 or 67 | 46172 |
| 69 | (systematic adj3 review$).ti,ab. | 4423 |
| 70 | 24 or 45 or 68 or 69 | 101114 |
| 71 | 10 and 19 and 70 | 218 |

**Table S6.**

Database: CINAHL PLUS

Host: EBSCOhost

Data Parameters: 1980-Current

Date of search: Wednesday 11^th^ December 2019

Search strategy:

| **#** | **Query** | **Results** |
| --- | --- | --- |
| S34 | S9 AND S17 AND S32 | 1,620 |
| S33 | S9 AND S17 AND S32 | 4,044 |
| S32 | S18 OR S19 OR S20 OR S21 OR S22 OR S23 OR S24 OR S25 OR S26 OR S27 OR S28 OR S29 OR S30 OR S31 | 995,078 |
| S31 | TI systematic review OR AB systematic review | 86,560 |
| S30 | TI ( (observational N1 (study or studies)) ) OR AB ( (observational N1 (study or studies)) ) | 43,313 |
| S29 | TI ( (cohort N1 (study or studies)) ) OR AB ( (cohort N1 (study or studies)) ) | 82,279 |
| S28 | (MM "Cross Sectional Studies") | 175 |
| S27 | (MM "Nonconcurrent Prospective Studies") | 292 |
| S26 | (MM "Nonconcurrent Prospective Studies") | 0 |
| S25 | (MM "Nonconcurrent Prospective Studies") | 292 |
| S24 | (MM "Nonconcurrent Prospective Studies") | 0 |
| S23 | (MM "Correlational Studies") | 57 |
| S22 | (MH "Case Control Studies+") | 75,582 |
| S21 | (MM "Prospective Studies") | 1,371 |
| S20 | TI random* OR AB random* | 308,099 |
| S19 | (MM "Qualitative Studies") | 3,207 |
| S18 | TI ( (Interview* or Experience* or qualitative) ) OR AB ( (Interview* or Experience* or qualitative) ) | 520,418 |
| S17 | S10 OR S11 OR S12 OR S13 OR S14 OR S15 OR S16 | 1,241,900 |
| S16 | TI ( ((treatment* OR Dialectical behavior therapy OR Dialectical behaviour therapy OR DBT OR Psychotherapy* OR specialist OR psychiatry* OR therapeutic OR day OR outreach OR therap* N3 (Outpatient* OR community OR Service* OR Center* OR Centre* OR Clinic* OR Team* OR program* OR provider* OR practice OR setting* OR care OR community OR unit* OR hospital*))) ) OR AB ( ((treatment* OR Dialectical behavior therapy OR Dialectical behaviour therapy OR DBT OR Psychotherapy* OR specialist OR psychiatry* OR therapeutic OR day OR outreach OR therap* N3 (Outpatient* OR community OR Service* OR Center* OR Centre* OR Clinic* OR Team* OR program* OR provider* OR practice OR setting* OR care OR community OR unit* OR hospital*))) ) | 1,073,764 |
| S15 | TI ( (network or outreach or ((specialist or day or whole) N3 service)) ) OR AB ( (network or outreach or ((specialist or day or whole) N3 service)) ) | 73,169 |
| S14 | TI ( (Independent sector or ((non institutional* or noninstitution*) N2 (sector* or setting*))) ) OR AB ( (Independent sector or ((non institutional* or noninstitution*) N2 (sector* or setting*))) ) | 416 |
| S13 | TI ( (commun* N5 (service or hub* or based or deliver* or interact* or led or maintenance or mediat* or operated or provides or provider* or run or setting* or support or rehab* or therap* or service* or treatment or management or assessment or assistance or care or day or week)) ) OR AB ( (commun* N5 (service or hub* or based or deliver* or interact* or led or maintenance or mediat* or operated or provides or provider* or run or setting* or support or rehab* or therap* or service* or treatment or management or assessment or assistance or care or day or week)) ) | 118,748 |
| S12 | TI ( (community N5 (agenc* or care or center* or centre* or clinic* or consultant* or doctor* or employee* or expert* or facilitator* or healthcare or instructor* or leader* or manager* or mentor* or nurs* or personnel* or pharmacy or pharmacist* or psychiatrist* or psychologist* or psychotherapist* or specialist* or skill* or staff* or team* or therapist* or tutor* or visit* or worker* or group* or independent or (peer* N3 support*) or survivor or outpatient* or "out patient*")) ) OR AB ( (community N5 (agenc* or care or center* or centre* or clinic* or consultant* or doctor* or employee* or expert* or facilitator* or healthcare or instructor* or leader* or manager* or mentor* or nurs* or personnel* or pharmacy or pharmacist* or psychiatrist* or psychologist* or psychotherapist* or specialist* or skill* or staff* or team* or therapist* or tutor* or visit* or worker* or group* or independent or (peer* N3 support*) or survivor or outpatient* or "out patient*")) ) | 71,566 |
| S11 | TI ( ((commun* N5 (mental health or model* or pathway* or program* or evaluat* or intervention* or implement*)) or camhs or cmht*) ) OR AB ( ((commun* N5 (mental health or model* or pathway* or program* or evaluat* or intervention* or implement*)) or camhs or cmht*) ) | 47,310 |
| S10 | (MM "Community Health Services") OR (MM "Community Mental Health Services") | 20,073 |
| S9 | S1 OR S2 OR S3 OR S4 OR S5 OR S6 OR S7 OR S8 | 26,480 |
| S8 | TI mood instability OR AB mood instability | 95 |
| S7 | TI ( (emotion* N2 (regulation or dysregulation or unstable or instability)) ) OR AB ( (emotion* N2 (regulation or dysregulation or unstable or instability)) ) | 4,493 |
| S6 | TI ( (Self-harm or self-injury) ) OR AB ( (Self-harm or self-injury) ) | 4,322 |
| S5 | TI ( (Complex N1 (needs or mental)) ) OR AB ( (Complex N1 (needs or mental)) ) | 3,104 |
| S4 | TI ( ("Complex trauma" or CPTSD or "complex post-traumatic stress disorder") ) OR AB ( ("Complex trauma" or CPTSD or "complex post- traumatic stress disorder") ) | 260 |
| S3 | TI "axis II" OR AB "axis II" | 492 |
| S2 | TI ( ((personality or character*) N3 disorder*) ) OR AB ( ((personality or character*) N3 disorder*) ) | 13,987 |
| S1 | (MM "Personality Disorders") | 2,867 |
