# Supplementary material for: Effectiveness of outpatient and community treatments for people with a diagnosis of ‘personality disorder’: systematic review and meta-analysis
Source: BMC Psychiatry. 2023 Jan 21;23:57. doi: 10.1186/s12888-022-04483-0 (PMC9862782; doi:10.1186/s12888-022-04483-0)
Supplement: Supplementary file 2 — Additional file 2. [file 12888_2022_4483_MOESM2_ESM.docx]

**
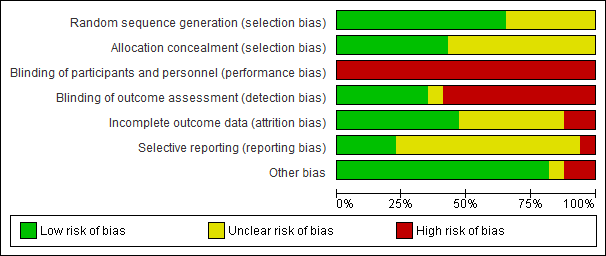
**

**Figure S1: Risk of bias graph: review authors' judgements about each risk of bias item presented as percentages across all included studies.**
