# Supplementary figures and images for: Effectiveness of outpatient and community treatments for people with a diagnosis of ‘personality disorder’: systematic review and meta-analysis
Source: BMC Psychiatry. 2023 Jan 21;23:57. doi: 10.1186/s12888-022-04483-0 (PMC9862782; doi:10.1186/s12888-022-04483-0)

**Figure S2: Funnel plots for publication bias.**


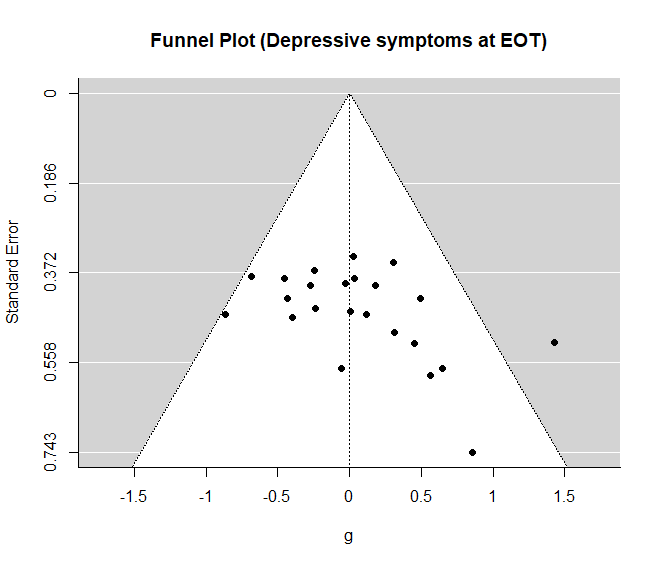

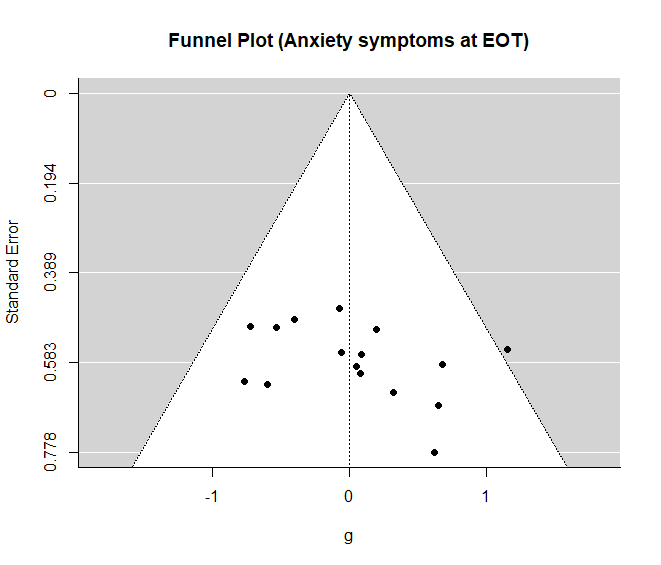

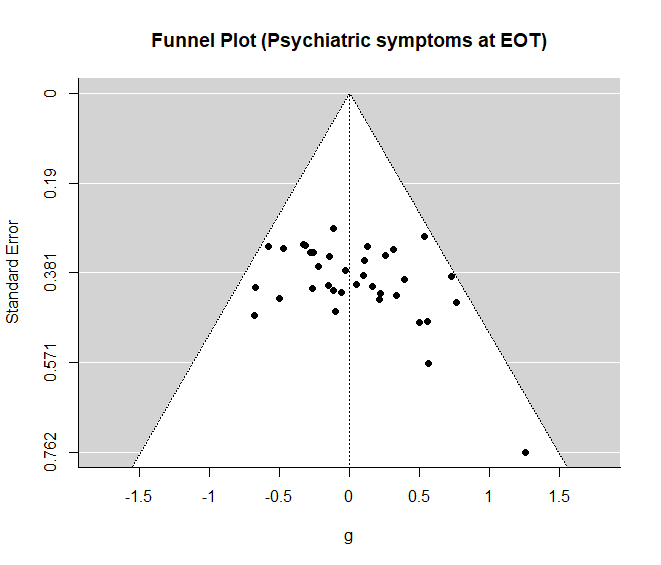

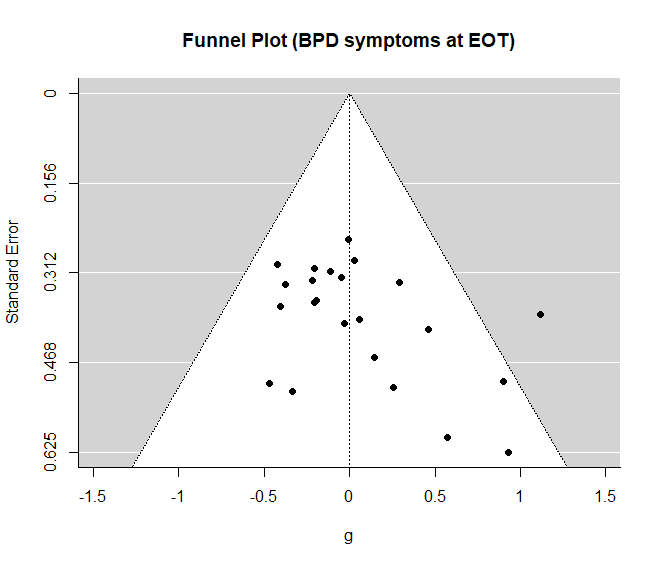

Supplement: Supplementary file 3 — Additional file 3. [file 12888_2022_4483_MOESM3_ESM.docx]
